# The VAX2-LINC01189-hnRNPF signaling axis regulates cell invasion and migration in gastric cancer

**DOI:** 10.1038/s41420-023-01688-4

**Published:** 2023-10-21

**Authors:** Linjie Hong, Ping Yang, Luyu Zhang, Xuehua Liu, Xiangyang Wei, Wushuang Xiao, Zhen Yu, Jieming Zhang, Ying Peng, Xiaosheng Wu, Weimei Tang, Fachao Zhi, Guoxin Li, Aimin Li, Jianjiao Lin, Side Liu, Hui Zhang, Li Xiang, Jide Wang

**Affiliations:** 1 Guangdong Provincial Key Laboratory of Gastroenterology, Department of Gastroenterology, Nanfang Hospital, Southern Medical University, Guangzhou, 510515 China; 2 Department of Gastroenterology, Shunde Hospital, Southern Medical University, Foshan, 528300 China; 3 Department of Gastroenterology, The Second Affiliated Hospital, School of Medicine, The Chinese University of Hong Kong, Shenzhen & Longgang District People’s Hospital of Shenzhen, Shenzhen, 518172 China; 4 Department of Gastroenterology, The Affiliated Hexian Memorial Hospital of Southern Medical University, Guangzhou, 511400 China

**Keywords:** Gastric cancer, Cell invasion, Transcriptional regulatory elements, Long non-coding RNAs

## Abstract

Transcription factors (TFs) and long noncoding RNAs (lncRNAs) contribute to gastric cancer (GC). However, the roles of TFs and lncRNAs in the invasion and metastasis of GC remain largely unknown. Here, we observed that the transcription factor VAX2 is significantly upregulated in GC cells and tissues and acts as an oncogene. Moreover, high VAX2 expression is associated with the advancement of tumors in GC. In terms of functionality, the enforced expression of VAX2 promotes the proliferation and metastasis of GC cells. Mechanistically, VAX2 specifically interacts with the LINC01189 promoter and represses LINC01189 transcription. Furthermore, LINC01189 exhibits significant downregulation in GC and functions as a suppressor gene. Functionally, it inhibits migratory and invasive abilities in GC cells. In the context of GC metastasis, VAX2 plays a role in modulating it by trans-repressing the expression of LINC01189. Additionally, LINC01189 binds to hnRNPF to enhance hnRNPF degradation through ubiquitination. The cooperation between LINC01189 and hnRNPF regulates GC cell invasion and migration. In addition, both VAX2 and hnRNPF are highly expressed, while LINC01189 is expressed in at low levels in GC tissues compared to normal gastric tissues. Our study suggests that VAX2 expression facilitates, while LINC01189 expression suppresses, metastasis and that the VAX2-LINC01189-hnRNPF axis plays a contributory role in GC development.

## Introduction

Gastric cancer (GC) is a significant contributor to mortality rates associated with neoplastic diseases. Approximately 40% of patients with GC have metastases, and only approximately 5% of these patients survive for 5 years [1]. Because of the absence of effective treatments, managing patients with metastatic GC is challenging [2, 3]. Therefore, to get insight into the molecular mechanisms of GC metastasis, it is crucial to identify the pivotal genes involving in GC metastasis.

The ventral anterior homeobox 2 (VAX2) gene is specifically expressed in the ventral region of the prospective neural retina in vertebrates [4–6]. A previous report indicated that VAX2 is elevated in papillary thyroid carcinoma (PTC) samples in comparison to normal thyroid tissues, and reducing VAX2 expression significantly inhibits the malignant biological behaviors of PTC cells [7]. The VAX2 gene encodes a protein that contains a homeodomain and is classified as a homeobox transcription factor [8]. Li et al. found that VAX2 regulates the expression of three genes (PLCB4, ADCY6, and CNR1) in breast cancer (BC) cells [9]. However, the pathophysiological roles and molecular mechanisms underlying GC remain elusive.

Long noncoding RNAs (lncRNAs) are transcripts that exceed 200 nucleotides in length but have limited or no protein-coding capacity. They have a significant impact on tumorigenesis, functioning as either tumor suppressors or oncogenes [10, 11]. For example, UPK1A-AS1 is significantly upregulated in liver cancer, thereby facilitating cellular proliferation and serving as an indicator of unfavorable prognosis [12]. Long intergenic noncoding RNA 00092 (LINC00092) exerts its effects on cancer-associated fibroblasts to promote glycolysis and progression of ovarian cancer (OC) [13]. LINC00284 is highly expressed and has been recognized as a potential target for gene therapy in tumors [14]. Specifically, the lncRNA PAXIP1-AS1 is transcriptionally repressed by HOXD9 to suppress GC cell proliferation and metastasis [15]. In addition, the lncRNAs NEAT1 and MALAT1 are transcriptionally activated by OCT4 to promote lung cancer progression [16]. LINC01189, a newly identified lncRNA, is highly conserved and present in various mammalian tissues. Recent studies have indicated that dysregulated LINC01189 may have prognostic significance in hepatocellular cancer (HCC) and BC [17, 18]. Nevertheless, the precise expression and functional mechanisms of LINC01189 in GC cells have yet to be determined.

Heterogeneous nuclear ribonucleoprotein F (hnRNPF), a member of the hnRNP family, is involved in gene expression and signal transduction [19, 20], playing significant roles in several types of cancer, including GC [21–25]. Honoré et al. showed that hnRNPF expression was elevated in GC tissues compared to normal tissues [23]. Furthermore, Li et al. revealed that hnRNPF showed a significant increase in BC tissues [24]. Importantly, Wang et al. showed that hnRNPF interacts with lncRNA-UCA1 to coregulate the malignant phenotypes of esophageal squamous cell carcinoma [25]. However, the relationship between hnRNPF and LINC01189 expression in GC has been poorly studied.

Here, we detected VAX2 and LINC01189 expression and investigated the biological functions and mechanisms of VAX2 and LINC01189 in GC. Moreover, the present work explored the association of LINC01189 with hnRNPF as well as the impact of LINC011896 in cooperation with hnRNPF on GC cell invasion and migration. Our study provided information on the VAX2-LINC01189-hnRNPF signaling axis in the progression of GC.

## Results

### VAX2 is overexpressed in GC cell lines and tissues

Bioinformatics analysis revealed that VAX2 was relatively highly expressed in normal human brain tissue but showed very low or no expression in normal tissues of the stomach, small intestine, duodenum, colon, liver, gallbladder, and other digestive organs, according to the GTEx database (https://www.gtexportal.org/home/, Supplementary Fig. 1A). However, it exhibited significant upregulation in most tumors compared to adjacent noncancerous normal tissues (FIREBROWSE database: http://firebrowse.org/ and UALCAN database: https://ualcan.path.uab.edu/, Supplementary Fig. 1B, C). Thus, VAX2 may be associated with different forms of cancer, including GC.

**Fig. 1:**
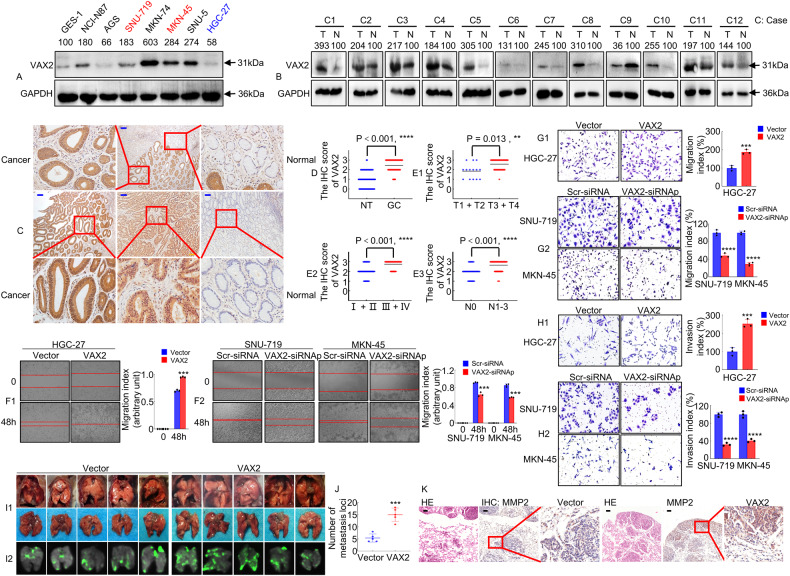
The expression and biological function analysis of VAX2 in GC cells and tissues. **A** VAX2 protein expression in normal gastric epithelial cell GES-1 and GC cell lines, including NCI-N87, AGS, SNU-719, MKN-74, MKN-45, SNU-5, and HGC-27, was detected through western blotting. Internal control was conducted using GAPDH. **B** A western blot showed the relative expression of VAX2 in 12 GC tissues compared with adjacent non-cancerous normal tissues. T, GC tissues: N, normal tissues. **C** Typical immunohistochemical images of VAX2 were obtained from a sample size of 74 GC tissues and their corresponding normal tissues. Scare bar: 100 µm. **D** VAX2 expression levels in different tissue groups from our study cohort. ****p < 0.001. **E**1-3 VAX2 expression levels in different subgroups stratified based on TNM stage, T stage, and N stage. Mann–Whitney U test, ****P < 0.001; **P < 0.05. **F**1/2 Migration potential of VAX2 overexpression (**F**1) or VAX2 knock-down (**F**2) GC cells in a wound healing assay. ***P < 0.01, Vector vs. VAX2; ***P < 0.01, Scr-siRNA vs. VAX2-siRNAp. Migration (**G**1/2) and invasion (**H**1/2) assays were performed on GC cells that were transfected with either Vector and VAX2 (**G**1 & **H**1) or Scr-siRNA and VAX2-siRNAp (**G**2 & **H**2). ***P < 0.01, Vector vs. VAX2; ****P < 0.001, Scr-siRNA vs. VAX2-siRNAp. **I**1/2 White-light (**I**1) and green fluorescence (**I**2) images of lung metastases in nude mice (*n* = 5). **J** Number of metastases in the lungs. ***P < 0.01, Vector vs. VAX2. **K** Representative images of H&E and IHC staining with antibodies against MMP2 in metastatic cancer tissue. Scale bar: 100 μm.

To detect VAX2 expression in GC, proteins from various GC cell lines were harvested and analyzed. We found that VAX2 protein levels were higher in the majority of GC cell lines, including NCI-N87, SNU-719, MKN-74, MKN-45, and SNU-5, than in GES-1 (an immortalized gastric mucosal epithelial cell line) (Fig. 1A). Then, we examined VAX2 expression in GC tissues via western blotting assays and observed that 10 out of 12 GC specimens exhibited elevated levels of VAX2 protein compared to their counterparts (Fig. 1B). For further validation, we measured VAX2 expression in matched cancerous (T) and normal (N) gastric tissues from 74 patients with GC using IHC analysis. Tumor cases usually displayed VAX2-positive staining, while adjacent normal cases were either VAX2-negative or weakly VAX2-positive (Fig. 1C). Semi-quantitative scoring showed that the expression of the VAX2 protein in cancer tissues was significantly higher than that in their counterpart tissues (Fig. 1D). Furthermore, the findings obtained from RNA sequencing data sourced from the TCGA database corroborated this observation (Supplementary Fig. 2A).

Next, we assessed the association between the expression of VAX2 and relevant clinicopathological factors to determine its clinical significance. The findings showed that increased VAX2 expression was significantly associated with differentiation (*P* = 0.03), AJCC T stage (T1/2 vs. T3/4, *P* = 0.013, Fig. 1E1), TNM stage (I/II vs. III/IV, *P* = 0.001, Fig. 1E2), and lymph node metastasis (*P* = 0.001, Fig. 1E3, N0 vs. N1-3). However, VAX2 is not correlated with extra clinical features, including age (*P* = 0.934), gender (*P* = 0.465), and tumor size (<10 cm^3^ vs. ≥10 cm^3^, *P* = 0.584) in GC (Supplementary Table 1). Because the follow-up period for the aforementioned samples was shorter than five years, we employed the expression and survival data from TCGA datasets to conduct a survival analysis on VAX2. Patients were categorized into groups with low and high expression based on the optimal threshold value. Kaplan–Meier survival curve analysis demonstrated a significant association between high VAX2 expression and reduced overall survival (OS, Supplementary Fig. 2B1) and disease-specific survival (DSS, Supplementary Fig. 2B2) in a considerable cohort of patients with GC (https://portal.gdc.cancer.gov/projects/).

Taken together, our data validated that VAX2 is upregulated in GC and positively associated with GC metastasis.

### VAX2 accelerates GC cell migration and invasion

To determine whether VAX2 had any effect on GC development and progression, we constructed stable transfectants using VAX2-sense plasmids in HGC-27 cells (Supplementary Fig. 2C1) or knocked down VAX2 with siRNAp in SNU-719 and MKN-45 cells (Supplementary Fig. 2C2), as confirmed through western blotting analysis.

First, to assess the impact of VAX2 on cell proliferation, EdU incorporation and colony formation assays were performed. Our findings indicated that the increased expression of VAX2 significantly promoted cellular growth in the EdU fluorescence staining assay (Supplementary Fig. 2D1). Moreover, overexpression of VAX2 in GC cells resulted in a markedly higher quantity of colonies when compared to the control vector (Supplementary Fig. 2E1). However, the growth and proliferation of GC cells were inhibited by the knockdown of VAX2 (Supplementary Fig. 2D2, E2).

Second, we investigated the effect of VAX2 on the migration and invasion of GC cells. Wound healing experiments showed that the increased expression of VAX2 enhanced the migration index of HGC-27 cells (Fig. 1F1), while producing the opposite results in SNU-719 and MKN-45 cells (Fig. 1F2). The transwell assays, with or without Matrigel, showed that the overexpression of VAX2 in HGC-27 cells resulted in increased migration and invasion abilities (Fig. 1G1, H1). Conversely, the knockdown of VAX2 in SNU-719 and MKN-45 cells resulted in reduced migratory and invasive activity compared to the control (Fig. 1G2, H2).

Third, an in vivo tail vein metastasis assay was performed on nude mice to assess the metastatic ability of VAX2 cells (Fig. 1I1/2). Notably, compared with HGC-27/Vector, inoculation of HGC-27/VAX2 cells led to a significant increase in visible tumors in the lungs, which was related to an increased number of metastatic loci (*P* < 0.01; Fig. 1J). An IHC assay was utilized to analyze the expression of MMP2 in lung metastases. The presence of lung metastases was confirmed through histological examination of lung sections stained with H&E, and the VAX2-overexpressing group exhibited a notable increase in MMP2 levels in GC tissues compared to the vector group in nude mice (Fig. 1K).

Together, these experiments collectively indicate that VAX2 facilitates the invasion and metastasis of GC cells.

### VAX2 binds specifically to VAX2-binding sites in the LINC01189 promoter

**Fig. 2:**
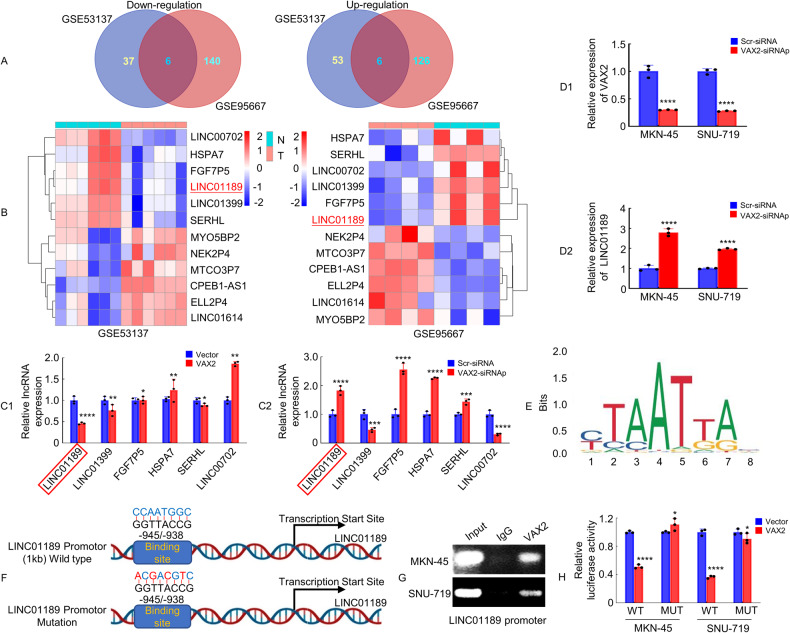
VAX2 binds specifically to VAX2-binding sites in the LINC01189 promoter. **A** Venn diagram displaying the differentially expressed lncRNA intersections between GC tissues and adjacent normal gastric tissues from GSE53137 and GSE95667 (https://www.ncbi.nlm.nih.gov/geo, threshold level: Log_2_FC = 1.5-fold). **B** Heatmap showing LINC00702, HSPA7, FGF7P5, LINC01189, LINC01399, and SERHL have low expression in GC tissues based on the GSE53137 and GSE95667 datasets. **C**1/2 Following overexpression (**C**1) or siRNA (**C**2) of VAX2 in HGC-27 cells, the expression of six selected lncRNAs was determined by qPCR. *P > 0.05; **P < 0.05; ****P < 0.001; Vector vs. VAX2. ***P < 0.01; ****P < 0.001; Scr-siRNA vs. VAX2-siRNAp. **D**1/2 Expression of VAX2 (**D**1) or LINC01189 (**D**2) in VAX2-knockdown MKN-45 and SNU-719 cells indicated by qPCR. ****P < 0.001, Scr-siRNA vs. VAX2-siRNAp. **E** Based on informatics analysis, the transcription factor VAX2-binding motif was predicted. **F** Schematic highlights the potential binding site in LINC01189 promotor for VAX2. **G** ChIP assays provided evidence of the interaction between VAX2 and the promoter region (−1 to −1 kb) of LINC01189. PCR and nucleic acid electrophoresis were performed to examine DNA fragments immunoprecipitated by anti-VAX2 or IgG antibody in MKN-45 and SNU-719 cells. **H** Luciferase reporter assays were employed to validate VAX2 binding to the LINC01189 promoter. WT Wild type, MUT Mutation. *P > 0.05 and ****P < 0.001, Vector vs. VAX2.

As a transcription factor, VAX2 may regulate downstream miRNAs [26], lncRNAs [15, 16], or protein-coding genes [8, 9]. To identify lncRNAs that showed significant differential expression (Log_2_FC = 1.5-fold) between GC tissues and normal gastric tissues, we obtained the GSE53137 and GSE95667 lncRNA microarray datasets. In the GSE53137 dataset, 43 lncRNAs were found to be downregulated and 59 were found to be upregulated, while in the GSE95667 dataset, 146 lncRNAs were downregulated and 132 were upregulated. According to the overlap between the two datasets, six annotated lncRNAs were downregulated, and six lncRNAs were upregulated (Fig. 2A). In Fig. 2B, blue represents downregulated (LINC00702, HSPA7, FGF7P5, LINC01189, LINC01399, and SERHL) and red represents upregulated (MYO5BP2, NEK2P4, MTCO3P7, CPEB1-AS1, ELL2P4, and LINC01614) lncRNAs. In this study, we explored whether VAX2 downregulates lncRNA transcription in GC cells.

We next evaluated whether the expression of a set of six molecules was influenced by the overexpression or knockdown of VAX2 in GC cells. We revealed that VAX2 upregulation significantly decreased the expression of LINC01189 and LINC01399 (Fig. 2C1), whereas VAX2 downregulation increased LINC01189, FGF7P5, HSPA7, and SERHL expression in HGC-27 cells (Fig. 2C2). The most notable alteration in LINC01189 expression was observed upon overexpression and knockdown of VAX2. Moreover, knockdown of VAX2 (Fig. 2D1) confirmed that VAX2 expression was negatively correlated with LINC01189 expression (Fig. 2D2) in MKN-45 and SNU-719 cells. We conducted additional research to examine the regulatory impact of VAX2 on LINC01189 expression in GC cells.

LINC01189 is located on human chromosome 9q13. According to the JASPAR database (Fig. 2E) [27], a putative VAX2 region is located 945–938 bp before the start of the transcriptional start site of LINC01189 (Fig. 2F). ChIP assays verified the direct interaction between VAX2 and the LINC01189 promoter in GC cells (Fig. 2G). Luciferase reporter assays demonstrated that the overexpression of VAX2 decreased the transcriptional activity of the LINC01189 promoter in GC cells. This effect was hindered by mutations in the VAX2-binding region, as indicated by the change in luciferase activity (Fig. 2H).

Collectively, this information indicates that VAX2 binds specifically to the VAX2-binding sites in the LINC01189 promoter, and plays a transcriptionally repressive role in LINC01189.

### LINC01189 is downregulated in GC cells and tissues

**Fig. 3:**
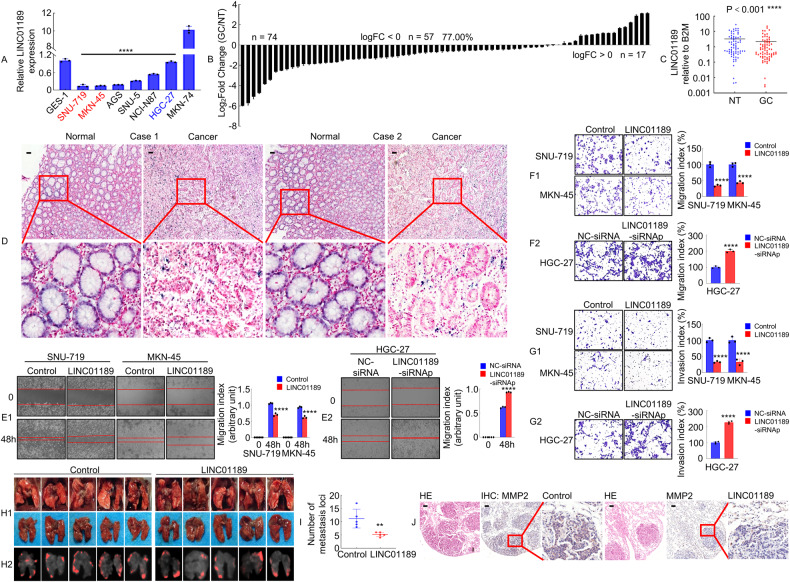
LINC01189 is downregulated in GC tissues and associated with GC metastasis. **A** Expression of LINC01189 was verified in GC cell lines and GES-1 cell line using qPCR. ****P < 0.001, GC cell lines vs. GES-1. **B** LINC01189 expression was assessed using qPCR in 74 paired GC tissues and adjacent normal tissues. **C** As analyzed by qPCR, the expression of LINC01189 in GC tissues was found to be significantly lower compared to adjacent normal tissues. ****P < 0.001. GC vs. NT. GC Gastric cancer, NT Normal tissues. **D** The localization and expression levels of LINC01189 in GC tissues and corresponding adjacent normal tissues (*n* = 30) were determined using in situ hybridization (ISH) analysis. Scale bar: 100 µm. **E**1/2 Migration potential of LINC01189 overexpression (**E**1) or LINC01189 knock-down (**E**2) in GC cells in a wound healing assay. ****P < 0.001, Control vs. LINC01189; ****P < 0.001, NC-siRNA vs. LINC01189-siRNAp. **F**1/2, **G**1/2 Migration (**F**1/2) and invasion (**G**1/2) transwell assays of GC cells transfected with LINC01189 overexpression plasmids (**F**1 & **G**1), LINC01189-siRNAp (**F**2 & **G**2), or the corresponding control for 48 h were conducted. **H**1/2 White-light (**H**1) and red fluorescence (**H**2) images of lung metastases in nude mice (*n* = 5). **I** Number of metastatic nodules in the lungs. **P < 0.05, Control vs. LINC01189. **J** Representative images of H&E and IHC staining with antibodies against MMP2 in metastatic cancer tissue. Scale bar: 100 μm.

To examine the expression of LINC01189, qPCR was performed using the following seven GC cell lines: SNU-719, MKN-45, AGS, SNU-5, NCI-N87, HGC-27, and MKN-74 and the immortalized GES-1. As shown in Fig. 3A, LINC01189 was downregulated in all GC cell lines, except MKN-74, compared to GES-1 cells. Additionally, qPCR was utilized to assess the expression of LINC01189 in 74 pairs of corresponding normal (N) and cancerous (T) gastric tissues. The findings suggested that LINC01189 was commonly downregulated in GC tissues compared to normal gastric mucosa (Fig. 3B, C). The expression level and subcellular localization of LINC01189 were analyzed by in situ hybridization (ISH) from a small cohort of 30 archived paraffin-embedded normal gastric and GC tissues. The findings showed that LINC01189 was localized in normal gastric tissues but was only marginally detectable in GC tissues (Fig. 3D). Consistent with the results of the ISH assay, subcellular fractionation experiments also indicated that LINC01189 was predominantly localized in the cell nucleus, with some presence in the cytoplasm (Supplementary Fig. 3A). To determine whether LINC01189 expression is involved in GC progression, we examined the association between LINC01189 and clinicopathological characteristics in 74 individuals diagnosed with GC. As illustrated in Supplementary Fig. 3B1–3 and Supplementary Table 2, statistical analysis revealed a correlation between LINC01189 expression and AJCC T stage (T1/2 vs. T3/4, *P* < 0.05, Supplementary Fig. 3B1), AJCC TNM stage (I/II vs. III/IV, *P* < 0.05, Supplementary Fig. 3B2), and lymph nodes metastasis (N0 vs. N1-3, *P* < 0.05, Supplementary Fig. 3B3). Kaplan–Meier curves showed that no notable distinction was observed between low- and high-level expression of LINC01189 in a limited number of GC patients from TCGA database (*P* > 0.05). However, high LINC01189 expression tended to reduce OS (Supplementary Fig. 3C).

Taken together, these results indicate that LINC01189 is downregulated in GC and is negatively associated with GC metastasis.

### LINC01189 inhibits the migration and invasion of GC cells

To ascertain whether LINC01189 is indeed a lncRNA [28, 29], the coding potential of this gene was evaluated utilizing the online analytical tools Coding Potential Assessment Tool (CPAT) and Coding Potential Calculator (CPC). The results showed a limited coding ability (Supplementary Fig. 3D). To determine whether LINC01189 was functional, overexpression or knockdown of LINC01189 was achieved by qPCR analysis (Supplementary Fig. 3E1-3).

To determine whether LINC01189 promotes GC cell development and progression, we conducted cell proliferation and transwell assays in vitro to evaluate the effects of overexpressing or knocking down LINC01189 in GC cells. An EdU assay showed that ectopic LINC01189 expression suppressed the growth of SNU-719 and MKN-45 cells (Supplementary Fig. 3F1). LINC01189 consistently suppressed colony formation by SNU-719 and MKN-45 cells (Supplementary Fig. 3G1). In contrast, LINC01189 knockdown significantly increased the growth rate of HGC-27 cells compared to the control group (Supplementary Fig. 3F2, G2). Moreover, wound-healing and transwell assays with or without Matrigel showed that ectopic LINC01189 expression was significantly inhibited, whereas LINC01189 knockdown promoted GC cell migration and invasion in vitro (Fig. 3E–G).

To examine the impact of LINC01189 on cellular metastasis in an in vivo setting, HGC-27/LINC01189 and HGC-27/control cells were introduced into the tail vein of nude mice to induce lung metastases (Fig. 3H1/2). The mice injected with LINC01189 cells exhibited a significantly reduced number of metastatic nodules in the lungs compared to those injected with control cells (Fig. 3I). Furthermore, IHC staining showed that the LINC01189-overexpressing group had decreased MMP2 expression compared to control cells in lung tissues (Fig. 3J). The histological analysis confirmed the existence of lung metastases derived from GC (Fig. 3J).

Our results reveal that LINC01189 suppresses the invasion and metastasis of GC cells.

### VAX2 modulated GC metastasis by transrepressing LINC01189 expression

**Fig. 4:**
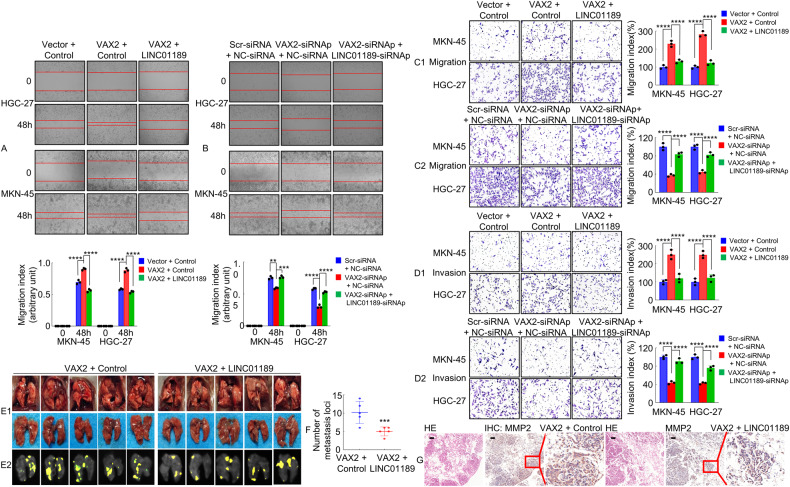
VAX2 modulates GC metastasis by transrepressing LINC01189 expression in GC cells. Migration potential of GC cells transfected with VAX2 and VAX2 + LINC01189 (**A**), or VAX2-siRNAp and VAX2-siRNAp + LINC01189-siRNAp (**B**) in a wound healing assay. **P < 0.05; ***P < 0.01; ****P < 0.001. Migration (**C**1/2) and invasion (**D**1/2) transwell assays of GC cells transfected with different treatments. Bar graphs on the right show the quantification of mobility capabilities of GC cells. ****P < 0.001. **E**1/2 White-light (**E**1) and yellow fluorescence (**E**2) images of lung metastases in nude mice (*n* = 5). **F** Number of metastases in the lungs. ***P < 0.01, VAX2 + Control vs. VAX2 + LINC01189. **G** Representative images of H&E and IHC staining with antibodies against MMP2 in metastatic cancer tissue. Scale bar: 100 μm.

To test whether LINC01189 is involved in VAX2-mediated GC invasion and metastasis, VAX2 or vector cells were transfected with LINC01189 or control. The results showed that overexpression of LINC01189 decreased the metastatic capacity of VAX2-enhanced GC cells (Fig. 4A, C1, D1), whereas LINC01189 knockdown partially reversed the mobility potential of VAX2-reduced GC cells (Fig. 4B, C2, D2).

To investigate the impact of LINC01189 on VAX2-mediated metastasis in an in vivo setting, HGC-27 cells expressing lentivirus (LV)-VAX2/Control or LV-VAX2/LINC01189 were introduced into the tail vein of nude mice to establish lung metastases (Fig. 4E1/2). Notably, LV-VAX2/LINC01189 cells resulted in a significant decrease in the quantity of observable metastatic tumors in the lungs, compared to the LV-VAX2/Control cell group (Fig. 4F). The presence of GC metastases in the lungs of nude mice and the difference in transfer ability between the two groups were further confirmed through HE and IHC staining using an anti-MMP2 antibody (Fig. 4G).

These results indicated that LINC01189 suppressed VAX2-enhanced metastasis in GC cells.

### LINC01189 binds to heterogeneous nuclear ribonucleoprotein F (hnRNPF) in human GC cells

**Fig. 5:**
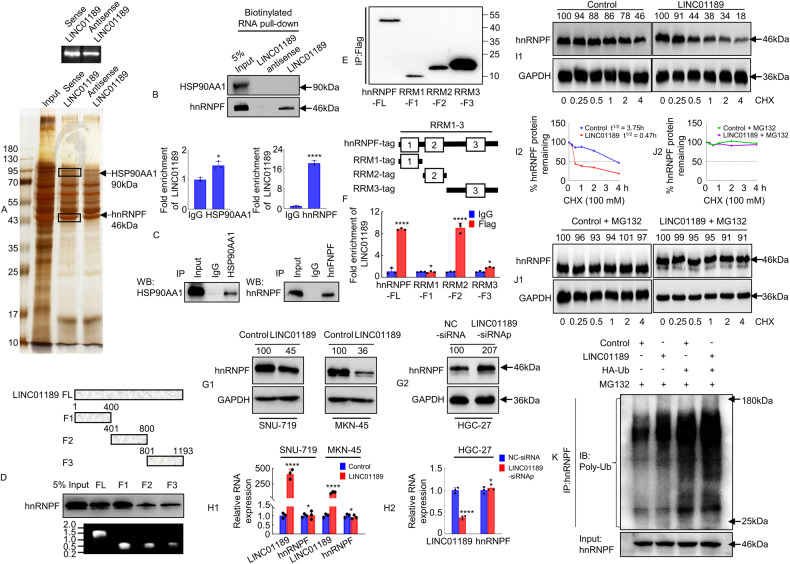
LINC01189 binds to hnRNPF in human GC cells. **A** Silver staining of the proteins binding to LINC01189. **B** RNA pulldown assay revealed specific binding of LINC01189 to hnRNPF, but not to HSP90AA1, in HGC-27 cells. **C** RNA immunoprecipitation (RIP) showed the specific interaction of hnRNPF, but not HSP90AA1, with LINC01189 in HGC-27 cells. *P > 0.05, IgG vs. HSP90AA1; ****P < 0.001, IgG vs. hnRNPF. **D** RNA pulldown assay analysis of hnRNPF binding to the truncations of LINC01189. Top: Schematic showing the LINC01189 truncations used. Bottom: Verification of RNA probes by nucleic acid electrophoresis. **E** Constructing and verification of full-length or truncated hnRNPFs plasmids using western blotting and anti-FLAG antibody. **F** RIP assay analysis of LINC01189 binding to the hnRNPF structure domains. Top: Schematic showing the hnRNPF structure domains used. ****P < 0.001, IgG vs. anti-hnRNPF-FL; *P > 0.05, IgG vs. anti-RRM1-F1 and IgG vs. anti-RRM3-F3; ****P < 0.001, IgG vs. anti-RRM2-F2. Error bars represent the mean ± SD from three independent experiments. **G**1/2 Total proteins from LINC01189 overexpression (**G**1) or siRNAp (**G**2) and control in GC cells were analyzed by western blotting to determine hnRNPF expression. **H**1/2 Total RNA from LINC01189 overexpression (**H**1) or siRNAp (**H**2) and control in GC cells analyzed using qPCR to determine hnRNPF and LINC01189 RNA levels. *P > 0.05 and ****P < 0.001, Control vs. LINC01189; *P > 0.05 and ****P < 0.001, NC-siRNA vs. LINC01189-siRNAp. Protein stability (**I**1/2) and degradation assays (**J**1/2) were performed for analysis of hnRNPF expression after transfection with LINC01189 or control and treatment with CHX (100 μM) and/or MG132 (10 μM) in HGC-27 cells. **K** The ubiquitin level of hnRNPF was measured using immunoprecipitation and western blotting. IP immunoprecipitation, IB immunoblotting.

Accumulating data have suggested that lncRNAs are directly associated with miRNAs, mRNAs, DNA, and proteins [30, 31]. To investigate the intricate mechanism by which LINC01189 controls the progression of GC, we sought to identify intracellular LINC01189-binding proteins. Biotinylated sense LINC01189 and antisense LINC01189 (serving as a negative control) were incubated with total protein extracts from HGC-27 cells and pulled down using streptavidin. SDS-PAGE and silver staining were utilized to analyze the protein products (Fig. 5A), followed by MS. The results showed that 65 proteins interacted with LINC01189, among which 11 proteins, including HS90A (HSP90AA1), HNRPF (HNRNPF), SF3B1, MYO1C, PYGB, RENT1 (UPF1), CUL4B, ALDOB, CUL4A, IQGA2, and TNFA, were associated with GC according to the PUBMED database (https://pubmed.ncbi.nlm.nih.gov/, 2023-6). HSP90AA1 and hnRNPF were the top two proteins enriched by in vitro transcribed biotinylated LINC01189, as determined by incorporating protein coverage and the exponentially modified protein abundance index (emPAI) (Supplementary Table 3). Western blotting analysis confirmed the presence of hnRNPF in the LINC01189 RNA pulldown complex, while HSP90AA1 was not detected (Fig. 5B). Furthermore, the RIP assay verified an endogenous interaction between LINC01189 and hnRNPF, but not HSP90AA1, in HGC-27 cells (Fig. 5C).

To identify the regions of binding, we utilized a series of LINC01189 truncations to map the hnRNPF-binding area. F1 represented the region from nucleotides 1–400 of LINC01189, which was the truncated 5′-end. F2 represented the region from nucleotides 401–800 of LINC01189, which was the truncated middle fragment. F3 represented the region from nucleotides 801–1193 of LINC01189, which was the truncated 3′-end. The findings suggested that hnRNPF interacts with either the F1, F2, or F3 region of LINC01189, mainly binding to the 5′-end. (Fig. 5D).

The hnRNPF protein is composed of three main domains: the RNA recognition motif (RRM) 1 to RRM3 domains [32, 33]. To further determine the LINC01189-binding region within the hnRNPF protein, we constructed hnRNPF (full-length), RRM1, RRM2, and RRM3 plasmids with a FLAG-tag (Fig. 5E). The in vitro binding assay indicated that RRM2, but not RRM1 or RRM3, is crucial for the interaction with LINC01189 (Fig. 5F). These results confirmed the specific binding of LINC01189 to hnRNPF in GC cells.

Given that numerous lncRNAs have demonstrated their ability to regulate the stability of binding proteins [30, 31, 34], our investigation delved deeper into whether the interaction of LINC01189 with hnRNPF affects protein stability. The findings indicated that overexpression of LINC01189 resulted in a decline in the protein expression of hnRNPF (Fig. 5G1) and vice versa (Fig. 5G2), but remained unaffected at the mRNA level (Fig. 5H1/2). The protein synthesis inhibitor CHX was used to treat both exogenous LINC01189 expressing and control cells, and subsequently, the protein level of hnRNPF expression was evaluated. The findings indicated that LINC01189 facilitated the degradation of hnRNPF (Fig. 5I1/2). To elucidate the mechanism by which LINC01189 mediates hnRNPF degradation, HGC-27 cells were subjected to MG132 treatment to inhibit protein degradation through the proteasome pathway. Subsequently, western blotting was conducted to compare the expression levels of hnRNPF between the LINC01189 group and the control group. The findings indicated that the proteasome inhibitor MG132 effectively reduced the degradation of hnRNPF caused by LINC01189 overexpression (Fig. 5J1/2). Furthermore, ubiquitination assays confirmed that LINC01189 promoted the ubiquitination of hnRNPF (Fig. 5K).

Collectively, our findings indicate that LINC01189 may directly interact with hnRNPF and enhance its degradation through ubiquitination, thereby regulating its expression.

### HnRNPF participates in LINC01189-mediated GC cell invasion and metastasis

**Fig. 6:**
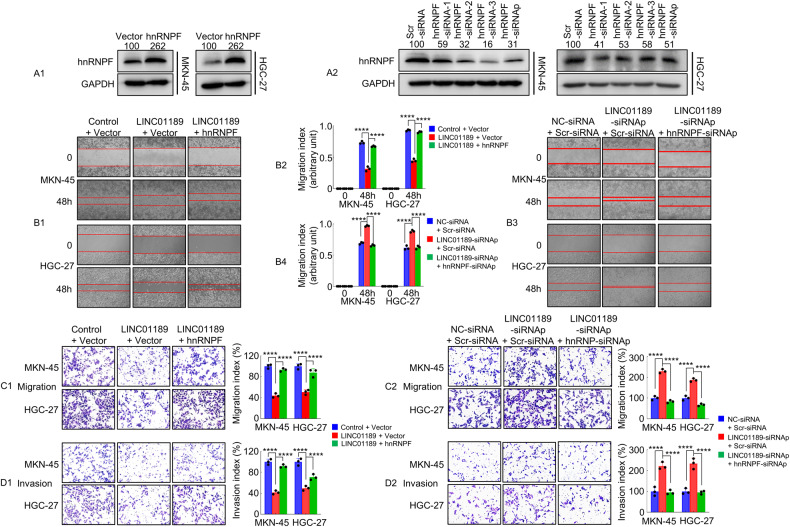
LINC01189 modulates hnRNPF-mediated GC invasion and metastasis in vitro. **A**1/2 Transfection efficiency of hnRNPF plasmid (**A**1) or hnRNPF siRNAs (**A**2) evaluated using western blotting in GC cells. **B**1-4 Migration potential of GC cells transfected with Control + Vector, LINC01189 + Vector, LINC01189 + hnRNPF (Left panel, **B**1), or NC-siRNA + Scr-siRNA, LINC01189-siRNAp + Scr-siRNA, LINC01189-siRNAp + hnRNPF-siRNAp (right panel, **B**3) in a wound healing assay. Quantitative results are shown in the center panels (**B**2 & **B**4). ****P < 0.001. Migration (**C**1/2) and invasion (**D**1/2) transwell assays of GC cells transfected with different treatments. ****P < 0.001.

To evaluate the possible association between LINC01189 and hnRNPF in GC cells, we first transfected cells with hnRNPF or vector (Fig. 6A1), hnRNPF-siRNAp or Scr-siRNA (Fig. 6A2). Western blotting was used to confirm the protein expression levels. We then conducted wound-healing and transwell assays. The findings indicated that the increased expression of LINC01189 suppressed cellular motility. However, overexpression of both LINC01189 and hnRNPF reversed this effect, and vice versa (Fig. 6B1/2, B3/4). Similarly, LINC01189 overexpression suppressed cell invasion and migration. In contrast, ectopic expression of LINC01189 cotransfected with the hnRNPF plasmid markedly reversed the outcome of LINC01189, and vice versa (Fig. 6C1/2, D1/2).

These findings demonstrated that LINC01189 cooperates with hnRNPF to regulate GC cell invasion and migration.

### Identification of a VAX2-LINC01189-hnRNPF axis in primary human GC samples

**Fig. 7:**
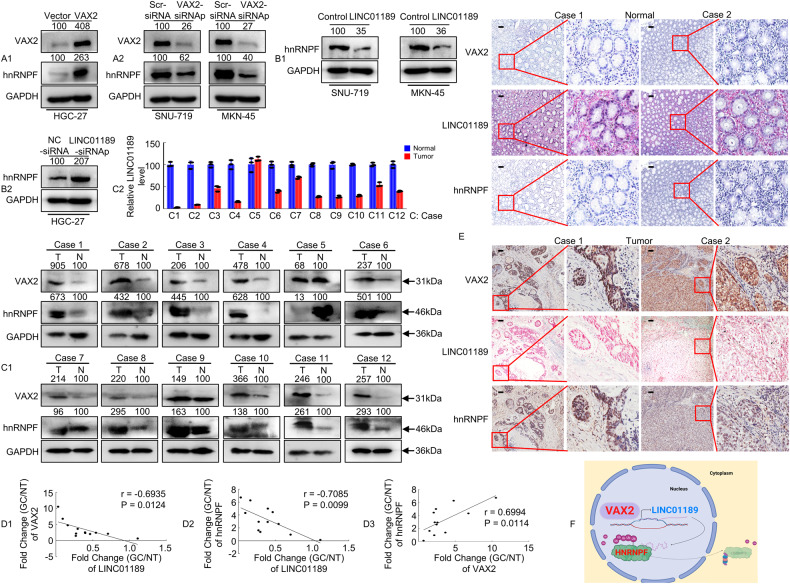
Identification of a VAX2-LINC01189-hnRNPF axis in primary human GC samples. **A**1/2, **B**1/2 VAX2 and/or hnRNPF expression detected using western blotting in GC cells transfected with Vector and VAX2 (**A**1), Scr-siRNA and VAX2-siRNAp (**A**2), Control and LINC01189 (**B**1), or NC-siRNA and LINC01189-siRNAp (**B**2). **C**1/2 The average tumor/normal epithelium (T/N) expression ratios of VAX2, hnRNPF, and LINC01189 detected using western blotting (**C**1) or qPCR (**C**2) in 12 paired GC tissues. C, case. **D**1-3 Correlation between VAX2 and LINC001189 expression levels, between LINC01189 and hnRNPF expression levels, or between VAX2 and hnRNPF expression levels in 12 GC tissues. Correlation coefficient (r) and *P*-value were acquired by Pearson correlation. **E** IHC and ISH were carried out to determine VAX2, LINC01189, and hnRNPF expression patterns in human GC tissues and normal epithelium tissues. Scale bar: 100 µm. **F** Proposed functional action of VAX2-LINC01189-hnRNPF axis in modulating GC progression.

To further investigate the relationships between VAX2 and hnRNPF, or LINC01189 and hnRNPF expression, the VAX2 plasmid or VAX2 siRNAp was transfected into GC cells for 48 h. The results demonstrated that upregulation of VAX2 significantly increased hnRNPF expression (Fig. 7A1), whereas downregulation of VAX2 (Fig. 7A2) decreased hnRNPF expression compared to that observed in vector cells. Additionally, we transfected GC cells with a LINC01189 plasmid, LINC01189 knockdown, or control at 48 h. Western blotting showed that LINC01189 overexpression (Fig. 7B1) decreased hnRNPF expression, whereas LINC01189 knockdown (Fig. 7B2) increased, hnRNPF expression relative to that of control cells.

To confirm the relationship among VAX2, LINC01189, and hnRNPF expression in primary GC tissues, we analyzed their expression levels in 12 matched clinical specimens from human GC patients. Using Western blotting or qPCR, we observed frequent upregulation of both VAX2 and hnRNPF expression (Fig. 7C1), while the expression of LINC01189 was found to be downregulated in the 12 GC samples compared to paired normal samples (Fig. 7C2). A negative correlation between VAX2 and LINC01189 was discovered through Spearman’s correlation analysis (Fig. 7D1), as well as between hnRNPF and LINC01189 (Fig. 7D2). There was a positive correlation between hnRNPF and VAX2 expression in GC tissues (Fig. 7D3). In addition, we performed IHC and ISH analyses and found that VAX2 or hnRNPF protein expression was negatively correlated with LINC01189 expression levels, whereas VAX2 expression was positively correlated with hnRNPF expression in the samples (Fig. 7E).

Collectively, our data demonstrate that the VAX2-LINC01189-hnRNPF axis may contribute to the progression of GC.

## Discussion

A number of studies have documented the dysregulated expression of transcription factor genes in diverse cancer types, including GC [7, 35, 36]. Previous studies have reported that proteins from transcription factor genes regulate lncRNA transcription during tumor progression [14, 15]. However, the regulation of lncRNA-LINC01189 by the transcription factor VAX2 in GC cells remains unexplored. In this study, we observed a significant upregulation of VAX2 and a notable downregulation of LINC01189 in GC tissues compared to normal tissues. Moreover, VAX2 overexpression stimulated, while LINC01189 overexpression suppressed, the proliferation and migration of GC cells. Furthermore, VAX2 trans-represses LINC01189 expression in GC cells. Additionally, LINC01189 binds to hnRNPF and participates in GC cell motility and invasion. Therefore, the VAX2-LINC01189-hnRNPF signaling axis regulates GC metastasis (Fig. 7F).

The VAX2 gene is situated on the short arm of human chromosome 2 region 2p13 and belongs to a subfamily of homeobox genes [4, 8, 37]. It is related the physiological processes in the vertebrate neural retina. Some studies have shown that it plays a role in the pathological mechanisms of various other illnesses. Norgett et al. reported that VAX2 may be associated with distal renal tubular acidosis [38]. Furthermore, the depletion of VAX2 significantly inhibits the proliferation, migration, and invasion of PTC cells [7]. However, the function and mechanism of VAX2 in GC remain unclear. In this study, VAX2 exhibited a significant increase in GC tissues compared with paired normal tissues according to the results of bioinformatics analysis, western blotting, and IHC assays. Moreover, overexpression of VAX2 in GC cells enhanced both cell proliferation and migration. Furthermore, VAX2 overexpression correlated with differentiation, advanced TNM stage, and positive lymph node metastasis. This finding aligns with our previously published research, wherein we demonstrated that the homeobox gene HOXD9 promotes the development and progression of GC cells [35]. These results suggest that VAX2 may function as an oncogene, facilitating the advancement and progression of GC.

LncRNAs have a crucial function in mediating gastric carcinogenesis and regulating GC progression. For instance, lncRNA-ZFPM2-AS1 promotes gastric carcinogenesis by stabilizing MIF [39], and lncRNA-CASC19 facilitates GC progression by preventing CREB1 protein degradation [40]. Regulation of lncRNA transcription could potentially involve transcription factors and epigenetic regulators [16, 41, 42]. For example, TEAD4, as a transcription factor, activates lncRNA-MNX1-AS1 and can accelerate the progression of GC via the EZH2/BTG2 and miR-6785-5p/BCL2 axes [42]. In this study, we demonstrated that VAX2 directly targets LINC01189 at the transcriptional level. The conclusions we have drawn are derived from the following observations: First, there was a negative correlation between the expression of VAX2 and LINC01189 in GC cells. Second, ChIP and luciferase reporter assays provided evidence of binding between VAX2 and the promoter of LINC01189. The promoter sequence from -945 to -938 bp was identified as the VAX2 response element (VAX2-RE). Third, mutation of VAX2-RE results in the blockage of VAX2 transcriptional activity. Consistent with our results, another report showed that STAT3 specifically interacts with the lncRNA-HOXD-AS1 promoter [43]. The findings indicate that VAX2 directly binds to and represses the LINC01189 promoter in GC cells.

LINC01189 is transcribed into a 1193-bp transcript and has recently been reported in several cancers. Yao et al. showed that LINC01189 overexpression inhibits HCC cell proliferation [17]. Moreover, LINC01189 inhibits BC progression by inhibiting EMT-like phenotypes [18]. However, the precise function of LINC01189 in GC remains poorly understood. In the current study, LINC01189 exhibited significant downregulation in GC cell lines and tissues. Furthermore, there was a notable association between its expression and unfavorable clinicopathological characteristics, including lymph node metastasis, AJCC T stage, and TNM stage. Functional experiments showed that LINC01189 effectively restrains the growth and metastasis/invasion of GC cells both in vitro and in vivo, which is consistent with reports that LINC01189 functions as a tumor suppressor gene [17, 18]. Given the significant contribution of LINC01189 to GC development and progression, our investigation aimed to explore whether LINC01189 is involved in VAX2-mediated GC metastasis. We observed that ectopic expression of VAX2 significantly increased the motility of GC cells, whereas LINC01189 overexpression decreased the migration potential of VAX2-overexpressing cells. In other words, LINC01189 overexpression blocked VAX2-mediated migration/invasion of GC and vice versa. Collectively, these findings indicate that VAX2 modulates GC metastasis by trans-repressing LINC01189 expression.

HnRNPF is located on chromosome region 10q11.21 and is involved in various cellular processes such as mRNA trafficking, stability, turnover, splicing regulation, and telomerase biogenesis [19, 20, 44, 45]. MS and coimmunoprecipitation studies have reported that hnRNPF interacts with TPX2 [21]. Furthermore, hnRNPF has the ability to directly bind to 3’UTR of the Snail1 mRNA, thereby enhancing its stability [22]. We discovered that LINC01189 might interact with hnRNPF through RNA pulldown, MS, and RIP assays. In addition, LINC01189 promoted hnRNPF degradation via ubiquitination in protein stability and degradation assays. Moreover, we employed functional assays and discovered that LINC01189 diminished the motility of GC cells, whereas hnRNPF reversed the LINC01189-mediated inhibition of invasion and migration in GC cells. Thus, we speculated that LINC01189 and hnRNPF coregulate GC cell motility. Additionally, IHC and ISH revealed a positive association between the expression of VAX2 protein and hnRNPF, whereas VAX2 or hnRNPF protein expression was negatively correlated with LINC01189 expression. This suggests that the VAX2-LINC01189- hnRNPF axis modulates GC progression.

To summarize, we introduce a new molecular foundation for the function of VAX2 in GC carcinogenesis, invasion, and metastasis. Moreover, VAX2 promotes the malignant phenotype of human GC cells via transrepression of LINC01189. Furthermore, LINC01189 inhibits the mobility of GC cells by binding to and destabilizing hnRNPF. Thus, targeting the VAX2-LINC01189-hnRNPF axis shows great potential as a therapeutic approach for treating GC.

## Materials and methods

### Human GC cell lines and reagents

Normal human gastric epithelial cells (GES-1) and GC cell lines, including NCI-N87, AGS, SNU-719, MKN-74, MKN-45, SNU-5, and HGC-27, were purchased from the American Type Culture Collection (Manassas, VA, USA). Cells were grown in base Roswell Park Memorial Institute medium (RPMI-1640, Thermo Fisher Scientific, USA) or Dulbecco’s Modified Eagle Medium (DMEM, Thermo Fisher Scientific, USA) supplemented with the addition of 10% fetal bovine serum (FBS, Biological Industries, Israel) and 100 U/mL penicillin/streptomycin solution (Sigma-Aldrich, USA) under standard conditions (5% CO_2_ and 37 °C) in humidified incubators.

### Western blot

Protein samples were obtained from either whole-cell lysates or GC tissues by utilizing RIPA Buffer and a protease inhibitor (100×) (Thermo Fisher Scientific, USA). Prior to being transferred to PVDF membranes (Merck Millipore, USA), the proteins were separation using sodium dodecyl sulfate-polyacrylamide gel electrophoresis (SDS-PAGE). 5% skim milk was used to block membranes at room temperature for 1 h, followed by overnight incubation with the primary antibody at 4 °C. After subsequent incubation of the membranes with secondary antibodies, protein levels were analyzed using ECL substrates (Beyotime, China). GAPDH served as an internal reference. Results were quantified using ImageJ 1.53. The antibodies used are listed in Supplementary Table 4.

### Clinical samples

74 formalin-fixed and paraffin-embedded GC samples and corresponding adjacent normal tissue samples were collected from GC patients at Nanfang Hospital (Guangzhou, China) between June 2017 and March 2020. Normal tissues were obtained at a distance of 5 cm from the tumor periphery, and all tissue samples were promptly preserved in liquid nitrogen for subsequent experimentation. The patients were diagnosed with primary GC by two pathologists and had not undergone any form of radiotherapy, chemotherapy, or molecular-targeted therapy prior to surgery. Ethical approval was obtained from Nanfang Hospital, Southern Medical University.

### Immunohistochemistry (IHC) and in situ hybridization (ISH) analyses

In GC specimens, IHC was used to detect VAX2 and hnRNPF expression. The extracted tissues were fixed in 10% formalin, dehydrated using ethanol, embedded in paraffin, and sliced into sections. The sections were heated at 65 °C for 2 h, dewaxed with xylene, rehydrated using gradient ethanol, treated with 3% hydrogen peroxide, heated in sodium citrate buffer (10 mM sodium citrate, 0.05% Tween-20, pH 6.0) for 15–20 min, blocked using goat serum for 20 min, and probed with primary antibody at room temperature for 2 h, followed by incubation with 50 μL secondary antibody for 1 h. Subsequently, the sections were subjected to a 5–10 min reaction with DAB (ZSGB-BIO, China), counterstained with hematoxylin for 2 min, and differentiated in hydrochloric acid (0.1 M). Following a 10-min rinsing period, the sections were dehydrated, cleared, and sealed for microscopic examination. The staining scores were determined by two independent pathologists. Scoring criteria: The staining intensity was graded on a scale of 0–3 (0 = negative, 1 = weak, 2 = moderate, and 3 = strong). The antibodies used are listed in Supplementary Table 4.

In GC specimens, ISH analysis was conducted to detect LINC01189 expression. Three oligonucleotide probes specific for LINC01189 were synthesized by BOSTER (Wuhan, China) as follows:

Probe 1: 5’ - AGTCCCCTGTGGTGACTGGAGGTTGGAGCCTGAAGATGGC - 3’;

Probe 2: 5’ - CAGGACTGCTGTGTCCAGAGTTGATTCCTTCTGGTGGGTT - 3’;

Probe 3: 5’ - ATGGATTGGTGCGTTTTACAGAGCGCTGATTGGTACATTT - 3’.

Briefly, paraffin-embedded sections were deparaffinized in xylene, rehydrated with gradient ethanol, washed with PBS, incubated with pepsin prepared in 3% sodium citrate at 37 °C for 10 min, fixed in 4% paraformaldehyde for 10 min, and added with nucleotide probes overnight at 42 °C. Next, stringent washing was performed using 2 × SSC, 0.5 × SSC, and 0.2 × SSC buffer at 37 °C for 15 min each. Anti-digoxigenin antibody (1:100; Roche, Germany) was then incubated at 37 °C for 1 h. Hybridization signals were visualized using nitro blue tetrazolium (NBT) and 5-bromo-4-chloro-3-indole phosphate (BCIP), and the reaction was terminated by washing with water for 5 min. Finally, the slides were counterstained with Nuclear Fast Red for 1 min and fixed in an aqueous solution.

### Plasmids, siRNAs assays, and cell transfection

His-VAX2, LINC01189, Flag-hnRNPF, HA-ubiquitin, wild-type or mutated promoter region of LINC01189, truncated LINC01189, and hnRNPF plasmids were provided by Kidan Biosciences (Guangzhou, China). siRNAs targeting VAX2, hnRNPF, or LINC01189 were chemically synthesized by Gene Pharma Company (Suzhou, China). The siRNA sequences are listed in Supplementary Table 6. Lipofectamine 3000 (Invitrogen, USA) was used to transiently transfect GC cells with these siRNAs.

### Quantitative PCR (qPCR)

Total RNA was extracted from GC cells and tissues using TRIzol reagent (Invitrogen, USA), according to the manufacturer’s instructions. The RNAs were reverse-transcribed into complementary DNAs (cDNAs) using a PrimeScript 1st Strand cDNA Synthesis Kit (Takara, Japan). The mRNA and lncRNA expression levels of the resulting cDNAs were measured using a SYBR Green PCR Kit (Takara, Japan) and a Roche LightCycler 480 system. Comparative quantification was performed using the 2^−ΔΔCt^ method. Human GAPDH or B2M was used as an internal control. The primer sequences are listed in Supplementary Table 5.

### Colony formation assay

Cells (5 × 10^2^ cells/well) were seeded in a 12-well plate and incubated for 14 days. After being fixed in 4% paraformaldehyde, colonies were visualized by staining the plates with 0.5% crystal violet. Colony formation rate was calculated using a microscope.

### 5-Ethynyl-2’-deoxyuridine (EdU) incorporation assay

An EdU Kit (RiboBio, Guangzhou, China) was used to monitor the proliferation of transfected cells. Concisely, GC cells were seeded in 96-well plates at a density of 2 × 10^4^ cells/well and incubated for 24 h. Cells were then treated with 50 µM EdU for 2 h at 37 °C. Subsequently, cells were fixed in 4% paraformaldehyde for 30 min and permeabilized with 0.5% Triton X-100 at room temperature for 10 min. Nuclei were counterstained with Hoechst 33342. The EdU incorporation assay results were visualized using an inverted fluorescence microscope (Olympus IX73, Japan) to capture five random fields. The EdU incorporation rate was defined as the ratio of EdU-positive cells (red) to total Hoechst 33342-positive cells (blue).

### Wound healing assay

GC cells transfected with various constructs were grown in six-well plates until they reached confluence. Wound injury was induced with the tip of a sterile micropipette and the detached cells were removed by washing with PBS. Cells were then incubated and allowed to migrate for 48 h. Photographs were taken and migration index was calculated as follows: migration index = (original wound width-wound width after healing)/original wound width × 100%. Each experiment was performed at least three times.

### In vitro transwell migration and invasion assay

In vitro migration and invasion assays were performed using 8-μm-pore transwell migration chambers (BD Biosciences, USA). The chambers were plated into a 24-well plate with 500 μL medium containing 10% FBS; GC cells (4 × 10^3^) resuspended in 200 μL serum-free medium were seeded into the upper chamber with (transwell invasion assay) or without (transwell migration assay) Matrigel (BD Biosciences, USA). After 48–72 h of incubation, the non-migrated cells were removed, the chambers were washed with PBS, fixed in 4% paraformaldehyde for 10 min, and stained with 0.5% crystal violet solution. The cells were counted in five random fields under a microscope. The migration and invasion rates in each group were determined by setting control samples to 100% migration/invasion.

### Promoter analysis and luciferase reporter assay

Promoters were defined as the 1.0 kb (−1000 bp to 0 bp) sequence upstream of the LINC01189 gene. The VAX2 binding sites (sequence:5’-CCAATGGC-3’, site: −945 to −938) in the LINC01189 promoter were predicted using JASPAR (http://jaspar.genereg.net/). Additional point mutations were introduced in the region of the VAX2 binding site (sequence:5’-ACGACGTC-3’, site: −945 to −938). GC cells were seeded in a 24-well plate and transfected with various plasmids. Firefly and Renilla luciferase activities were measured using the Duo-Lite Luciferase Assay System (Vazyme, China), and firefly luciferase activity was normalized to Renilla luciferase activity. Each experiment was performed at least three times.

### Chromatin immunoprecipitation (ChIP) assays

Briefly, GC cells (1 × 10^7^) were treated with 1% formaldehyde for 10 min at room temperature to induce DNA-protein cross-linking. Chromatin was sonicated to fragments ranging from 200 to 800 bp following the protocol provided by the SimpleChIP Enzymatic Chromatin IP Kit (Cell Signaling, USA). Next, the chromosome fragments were captured using bead-antibody complexes, washed, eluted, and purified. Finally, PCR was used to detect the fragments of the LINC01189 gene promoter containing the predicted VAX2 binding site. The antibodies used are listed in Supplementary Table 4 and the primer sequences are listed in Supplementary Table 5.

### Coding capacity analysis

The Coding Potential Calculator 2 (CPC2, http://cpc2.gao-lab.org/) and Coding Potential Assessment Tool (CPAT, http://lilab.research.bcm.edu/cpat/) were used to assess the coding capacity of LINC01189. GAPDH was used as a representative protein-coding gene, whereas NEAT1 was selected as a proxy for non-coding genes.

### Subcellular fractionation

We used a cytoplasmic and nuclear RNA purification kit (Norgenbiotek, Canada) to isolate cytoplasmic and nuclear RNA from human GC cells, according to the instructions provided by the manufacturer. RNA was reverse-transcribed to cDNA, and qPCR was performed to examine its subcellular localization. Quality control of cytoplasmic RNA was conducted using GAPDH, and quality control of nuclear RNA was conducted using NEAT1. The primer sequences are listed in Supplementary Table 5.

### RNA pulldown and mass spectrometry (MS)

Biotin-labeled RNA probes were prepared with the RNAmax-T7 in vitro transcription kit (RiboBio, China). Reactions were carried out at 37 °C for 2 h, and template DNA was removed by treatment with DNase I. After purification, magnetic beads (Bersin Bio, China) were added to the binding RNA probes, incubated with cell lysates, and eluted with elution buffer. MS or western blotting was used to analyze a fraction of the eluted proteins, which were separated by SDS-PAGE. LC-MS/MS was performed by FitGene Bio Company (Guangzhou, China). All MS results are listed in Supplementary Table 3.

### RNA immunoprecipitation (RIP) assay

The Magna RIP Kit (Merck Millipore, USA) was utilized for the RIP assay. Prior to immunoprecipitation, magnetic beads were bound with 5 μg specific primary antibodies for 30 min at room temperature. Subsequently, the complexes of beads and antibodies were incubated with GC cell lysates for 12 h at 4 °C. RNA was extracted, reverse-transcribed, and quantified using qPCR. HGC-27 cells were transfected with full-length or truncated FLAG-hnRNPF plasmids. Cell lysates were prepared for anti-FLAG antibody RIP as described above. The antibodies used are listed in Supplementary Table 4 and the primer sequences are listed in Supplementary Table 5.

### Protein stability, degradation and ubiquitination assay

Protein stability assays were performed using cycloheximide (CHX; Selleck, China).

After transfection with the indicated LINC01189 or control plasmids, GC cells were treated with 100 μM CHX for0, 0.25, 0.5, 1, 2, or 4 h. Total cellular proteins were extracted and quantified using a bicinchoninic acid (BCA; Thermo Fisher Scientific, USA) assay. The abundance of endogenous hnRNPF was calculated by western blotting using ImageJ 1.53.

Carbobenzoxy-L-leucyl-L-leucyl-L-leucinal (MG132; Selleck, China), a 26 S proteasome inhibitor, was used for protein degradation assays. In addition to treatment with plasmids and 100 μM CHX, HGC-27 cells were also incubated with 10 μM MG132 for 6 h before lysis. The level of hnRNPF degradation was estimated using western blotting. The expression levels of hnRNPF in the degradation assays were confirmed by western blotting analysis.

A ubiquitination assay was conducted to detect ubiquitinated hnRNPF. First, cells were transfected with empty, LINC01189, or HA-ubiquitin plasmids for 48 h. After 6 h of treatment with 10 μM MG132, total proteins were prepared and immunoprecipitated with the anti-hnRNPF antibody on protein A/G beads (Selleck, China) overnight at 4 °C. Proteins were washed and separated in 10% SDS-PAGE gel. Ubiquitinated hnRNPF was detected using an antibody against ubiquitination, and grayscale analysis was used to quantify the ubiquitinated protein levels.

### Lentiviral infection assay

The lentiviral constructs were purchased from Obio Technology (Shanghai, China). The detailed constructs are as follows: pSLENti-vector (pSLENti-EF-1-EGFP-F2A-Puro-CMV-MCS-WPRE), pSLENti-VAX2 (pSLENti-EF-1-EGFP-F2A-Puro-CMV-VAX2-3×flag-WPRE), pLENti-Control (pLENti-EF1-mCherry-P2A-Neo-CMV-MCS-3×flag-WPRE), and pLENti-Linc01189(pLENti-EF1-mCherry-P2A-Neo-CMV-MCS-Linc01189). GC cell lines were transduced with lentiviruses by adding 5 μg/mL polybrene. Expression of these genes in isolated cells was confirmed via western blot or qPCR after selection with G418 or puromycin. Selected pools of confirmed overexpressing cells were used for subsequent studies.

### Animal studies

Female BALB/c nude mice (nu/nu, aged 4–6 weeks) were purchased from Guangdong Medical Laboratory Animal Center (China) and maintained in specific pathogen-free facilities located at Southern Medical University (China). The animal experimental protocols used in this study were approved by the Animal Ethics Committee of Nanfang Hospital.

For the lung metastasis model, nude mice were randomly divided into different groups (5 mice per group). The stably transfected HGC-27 cell suspension (5 × 10^6^ cells suspended in 0.1 mL PBS per mouse) was injected into the tail veins of nude mice, which were euthanized and dissected on day 42. The number of metastatic loci in each lung tissue sample was examined using fluorescence imaging. Lung tissues were tested for bioluminescence as previously described, and then fixed in 10% formalin. Paraffin sections (3 mm) were prepared for hematoxylin and eosin (HE) staining and immunochemistry using an anti-MMP2 antibody.

### Statistical analysis

Statistical analyses were conducted using SPSS version 23.0 software (SPSS Inc., USA) and GraphPad Prism 8 (GraphPad Software, USA). Quantitative data are presented as the mean ± standard deviation (SD). For normally distributed continuous variables, Student’s *t* test or one-way analysis of variance (ANOVA) was used where appropriate, whereas for other comparisons between qualitative variables, the Mann–Whitney U test was applied. The frequency table (Supplementary Table 1) was analyzed using the chi-square test. Statistical significance was defined as a two-tailed *P* < 0.05. All in vitro experiments were conducted three times.

### Supplementary information

Supplementary Tables

Supplementary Fig. 1

Supplementary Fig. 2

Supplementary Fig. 3

Supplementary Figure legends

Supplementary File Raw data of western blotting

## Data Availability

All data are available within the article and supplementary files, or from the authors upon reasonable request.
