# Supplementary material for: The VAX2-LINC01189-hnRNPF signaling axis regulates cell invasion and migration in gastric cancer
Source: Cell Death Discov. 2023 Oct 21;9:387. doi: 10.1038/s41420-023-01688-4 (PMC10590441; doi:10.1038/s41420-023-01688-4)
Supplement: Supplementary file 1 — Supplementary Tables [file 41420_2023_1688_MOESM1_ESM.docx]

**Supplementary tables**

**Supplementary Table 1. Correlation between VAX2 protein expression and the clinicopathological parameters of GC**

| Features | Total  number  (n=74) | VAX2 Expression | | P |
| --- | --- | --- | --- | --- |
|  |  | Low  (Score 0-1) | High  (Score 2-3) |  |
| Age (years) |  |  |  | 0.934 |
| <60 | 33 | 14 | 19 |  |
| ≥60 | 41 | 17 | 24 |  |
| Gender |  |  |  | 0.465 |
| Male | 54 | 24 | 30 |  |
| Female | 20 | 7 | 13 |  |
| Tumor size (cm3) |  |  |  | 0.584 |
| <10 | 48 | 19 | 29 |  |
| ≥10 | 26 | 12 | 14 |  |
| Differentiation |  |  |  | 0.030 |
| Well/Moderate | 17 | 11 | 6 |  |
| Poor | 57 | 20 | 37 |  |
| AJCC stage |  |  |  | 0.013 |
| T1/T2 | 14 | 10 | 4 |  |
| T3/T4 | 60 | 21 | 39 |  |
| Lymph nodes (N) |  |  |  | 0.001 |
| Absent (N0) | 22 | 18 | 4 |  |
| Present (N1-3) | 52 | 13 | 39 |  |
| AJCC TNM stage |  |  |  | 0.001 |
| I/II | 32 | 23 | 9 |  |
| III/IV | 42 | 8 | 34 |  |

**Supplementary Table 2. Correlation between LINC01189 expression and clinicopathological parameters of GC**

| Features | Total number (n=74) | LINC01189 Expression | P |
| --- | --- | --- | --- |
| Age (years) |  |  | 0.175 |
| <60 | 33 | 1.441±2.106 |  |
| ≥60 | 41 | 0.871±1.240 |  |
| Gender |  |  | 0.627 |
| Male | 54 | 1.184±1.931 |  |
| Female | 20 | 0.967±0.758 |  |
| Tumor size (cm3) |  |  | 0.205 |
| <10 | 48 | 1.168±1.687 |  |
| ≥10 | 26 | 0.797±0.794 |  |
| Differentiation |  |  | 0.275 |
| Well/Moderate | 17 | 1.720±2.662 |  |
| Poor | 57 | 0.970±1.256 |  |
| AJCC stage |  |  | 0.048 |
| T1/T2 | 14 | 2.657±3.223 |  |
| T3/T4 | 60 | 0.768±0.757 |  |
| Lymph nodes(N) |  |  | 0.012 |
| Absent (N0) | 22 | 2.252±2.711 |  |
| Present (N1-3) | 52 | 0.648±0.546 |  |
| AJCC TNM stage |  |  | 0.010 |
| I/II | 32 | 1.786±2.340 |  |
| III/IV | 42 | 0.621±0.603 |  |

**Supplementary Table 3.** Mass spectra obtained for LINC01189 pulldown proteins

| Gene name | UniProtKB | Description | score | mass | matches | emPAI |
| --- | --- | --- | --- | --- | --- | --- |
| ACTG1 | P63261 | Actin, cytoplasmic 2 | 169 | 42108 | 10 | 0.98 |
| HSP90AA1^*^ | P07900 | Heat shock protein HSP 90-alpha | 159 | 85006 | 4 | 0.16 |
| QARS1 | P47897 | Glutamine--tRNA ligase | 51 | 88655 | 1 | 0.04 |
| TUBA8 | Q9NY65 | Tubulin alpha-8 chain | 50 | 50746 | 2 | 0.13 |
| MARS1 | P56192 | Methionine--tRNA ligase, cytoplasmic | 47 | 102249 | 1 | 0.03 |
| TUBB4A | P04350 | Tubulin beta-4A chain | 41 | 50010 | 4 | 0.29 |
| HNRNPF^*^ | P52597 | Heterogeneous nuclear ribonucleoprotein F | 40 | 45985 | 2 | 0.15 |
| AARS2 | Q5JTZ9 | Alanine--tRNA ligase, mitochondrial | 36 | 108299 | 1 | 0.03 |
| SF3B1^*^ | O75533 | Splicing factor 3B subunit 1 | 32 | 146479 | 2 | 0.05 |
| DPEP1 | P16444 | Dipeptidase 1 | 30 | 46101 | 1 | 0.07 |
| USO1 | O60763 | General vesicular transport factor p115 | 29 | 108740 | 1 | 0.03 |
| F11 | P03951 | Coagulation factor XI | 29 | 72116 | 1 | 0.05 |
| RAD21 | O60216 | Double-strand-break repair protein rad21 homolog | 29 | 71930 | 1 | 0.05 |
| TTLL3 | Q9Y4R7 | Tubulin monoglycylase TTLL3 | 29 | 88612 | 1 | 0.04 |
| TRMT10C | Q7L0Y3 | tRNA methyltransferase 10 homolog C | 28 | 47602 | 1 | 0.07 |
| CHD3 | Q12873 | Chromodomain-helicase-DNA-binding protein 3 | 28 | 227989 | 1 | 0.01 |
| MYO1C^*^ | O00159 | Unconventional myosin-Ic | 28 | 122461 | 1 | 0.03 |
| SCN5A | Q14524 | Sodium channel protein type 5 subunit alpha | 28 | 229188 | 1 | 0.01 |
| SMC3 | Q9UQE7 | Structural maintenance of chromosomes protein 3 | 27 | 141853 | 1 | 0.02 |
| ANKAR | Q7Z5J8 | Ankyrin and armadillo repeat-containing protein | 27 | 163804 | 1 | 0.02 |
| AMOT | Q4VCS5 | Angiomotin | 27 | 118469 | 1 | 0.03 |
| ZW10 | O43264 | Centromere/kinetochore protein zw10 homolog | 27 | 89628 | 1 | 0.04 |
| PYGB^*^ | P11216 | Glycogen phosphorylase, brain form | 27 | 97319 | 1 | 0.03 |
| POLDIP3 | Q9BY77 | Polymerase delta-interacting protein 3 | 27 | 46289 | 2 | 0.15 |
| SIGLEC16 | A6NMB1 | Sialic acid-binding Ig-like lectin 16 | 27 | 53757 | 1 | 0.06 |
| PTPDC1 | A2A3K4 | Protein tyrosine phosphatase domain-containing protein 1 | 27 | 85543 | 1 | 0.04 |
| UPF1^*^ | Q92900 | Regulator of nonsense transcripts 1 | 26 | 125578 | 1 | 0.03 |
| CUL4B^*^ | Q13620 | Cullin-4B | 26 | 104486 | 1 | 0.03 |
| SRRT | Q9BXP5 | Serrate RNA effector molecule homolog | 26 | 101060 | 1 | 0.03 |
| TMPPE | Q6ZT21 | Transmembrane protein with metallophosphoesterase domain | 26 | 49706 | 1 | 0.07 |
| PFKP | Q01813 | ATP-dependent 6-phosphofructokinase, platelet type | 26 | 86454 | 1 | 0.04 |
| ALDOB^*^ | P05062 | Fructose-bisphosphate aldolase B | 26 | 39961 | 1 | 0.08 |
| NUP155 | O75694 | Nuclear pore complex protein Nup155 | 26 | 156697 | 1 | 0.02 |
| SUPT5H | O00267 | Transcription elongation factor SPT5 | 25 | 121324 | 1 | 0.03 |
| IL4R | P24394 | Interleukin-4 receptor subunit alpha | 25 | 91596 | 1 | 0.04 |
| MARCHF9 | Q86YJ5 | E3 ubiquitin-protein ligase MARCHF9 | 25 | 38490 | 1 | 0.09 |
| HADHA | P40939 | Trifunctional enzyme subunit alpha, mitochondrial | 25 | 83688 | 1 | 0.04 |
| DOCK1 | Q14185 | Dedicator of cytokinesis protein 1 | 25 | 216178 | 1 | 0.02 |
| CEP95 | Q96GE4 | Centrosomal protein of 95 kDa | 25 | 95865 | 1 | 0.03 |
| CUL4A^*^ | Q13619 | Cullin-4A | 25 | 88138 | 1 | 0.04 |
| GRM1 | Q13255 | Metabotropic glutamate receptor 1 | 25 | 134154 | 1 | 0.02 |
| TSNAX | Q99598 | Translin-associated protein X | 24 | 33206 | 1 | 0.1 |
| TBC1D8B | Q0IIM8 | TBC1 domain family member 8B | 24 | 130110 | 1 | 0.03 |
| RP1L1 | Q8IWN7 | Retinitis pigmentosa 1-like 1 protein | 24 | 254474 | 1 | 0.01 |
| PLEC | Q15149 | Plectin | 24 | 533462 | 1 | 0.01 |
| MTHFD1L | Q6UB35 | Monofunctional C1-tetrahydrofolate synthase, mitochondrial | 24 | 106636 | 1 | 0.03 |
| CERK | Q8TCT0 | Ceramide kinase | 24 | 61194 | 1 | 0.05 |
| EPC1 | Q9H2F5 | Enhancer of polycomb homolog 1 | 23 | 93975 | 1 | 0.03 |
| UACA | Q9BZF9 | Uveal autoantigen with coiled-coil domains and ankyrin repeats | 23 | 163545 | 1 | 0.02 |
| PDS5B | Q9NTI5 | Sister chromatid cohesion protein PDS5 homolog B | 23 | 165818 | 1 | 0.02 |
| EEF1A1P5 | Q5VTE0 | Putative elongation factor 1-alpha-like 3 | 23 | 50495 | 1 | 0.07 |
| ERCC6L2 | Q5T890 | DNA excision repair protein ERCC-6-like 2 | 23 | 178955 | 1 | 0.02 |
| TBL1X | O60907 | F-box-like/WD repeat-containing protein TBL1X | 22 | 63255 | 1 | 0.05 |
| TSSK6 | Q9BXA6 | Testis-specific serine/threonine-protein kinase 6 | 22 | 30654 | 1 | 0.11 |
| IQGAP2^*^ | Q13576 | Ras GTPase-activating-like protein IQGAP2 | 22 | 181036 | 1 | 0.02 |
| SEC23A | Q15436 | Protein transport protein Sec23A | 22 | 87018 | 1 | 0.04 |
| GDE | P35573 | Glycogen debranching enzyme | 22 | 176819 | 1 | 0.02 |
| TNF^*^ | P01375 | Tumor necrosis factor | 20 | 25856 | 1 | 0.13 |
| ADCY10 | Q96PN6 | Adenylate cyclase type 10 | 20 | 189592 | 1 | 0.02 |
| IKZF4 | Q9H2S9 | Zinc finger protein Eos | 18 | 65263 | 1 | 0.05 |
| ZNF142 | P52746 | Zinc finger protein 142 | 18 | 193289 | 1 | 0.02 |
| PIGG | Q5H8A4 | GPI ethanolamine phosphate transferase 2 | 18 | 109187 | 1 | 0.03 |
| PPIG | Q13427 | Peptidyl-prolyl cis-trans isomerase G | 18 | 89077 | 1 | 0.04 |
| MIB2 | Q96AX9 | E3 ubiquitin-protein ligase MIB2 | 17 | 111638 | 1 | 0.03 |
| AGO4 | Q9HCK5 | Protein argonaute-4 | 16 | 98175 | 1 | 0.03 |

^*^, The proteins labeled as blue were found to be associated with GC according to the PUBMED database (https://pubmed.ncbi.nlm.nih.gov/, 2023-6)

**Supplementary Table 4. List of antibodies used in this study**

| **Antibody** | **Vendor** | **Catalog No.** | **Application** |
| --- | --- | --- | --- |
| VAX2 | Proteintech | 15773-1-AP | WB: 1:1000  IHC: 1:50 |
| VAX2 | Santa Cruz Biotechnology | sc-81422 | ChIP: 2μg |
| hnRNPF | Santa Cruz Biotechnology | sc-32309 | IP: 2μg  RIP:5μg |
| hnRNPF | Proteintech | 14974-1-AP | WB: 1:1000  IHC: 1:100 |
| HSP90AA1 | ABclonal | A5006 | WB: 1:1000  RIP:5μg |
| Ubiquitin | Proteintech | 10201-2-AP | WB: 1:1000 |
| GAPDH | Proteintech | 60004-1-Ig | WB: 1:30000 |
| Flag | Proteintech | 66008-4-Ig | WB: 1:2000  IP: 2μg  RIP:5μg |

**Supplementary Table 5. List of primers used in this study**

| **Experiment** | **Name** | **Position or orientation** | **Sequence (5’-3’)** |
| --- | --- | --- | --- |
| qPCR | VAX2 | F | AGCGGACACGTACATCCTTC |
|  |  | R | CAGACCTTCACCTGGGTCTC |
|  | LINC01189 | F | AACAATCTACAATAGCTGGCATT |
|  |  | R | AACAAAGGTAGTAAAATTCCTGGG |
|  | hnRNPF | F | ACATTTACAACTTCTTCTCTC |
|  |  | R | AACTCAACATCTGCTTCA |
|  | GAPDH | F | AAATCCCATCACCATCTTCC |
|  |  | R | TCACACCCATGACGAACA |
|  | B2M | F | ACTGAATTCACCCCCACTGA |
|  |  | R | CCTCCATGATGCTGCTTACA |
|  | NEAT1 | F | TGTGGTGGTGGGTGCCTGTAG |
|  |  | R | TGACTGTAACCTCCGCCTCCTG |
|  | SERHL | F | CCAAGTCCCTAAGCGGTTCC |
|  |  | R | GCGTGTGTGTGCACTGTAAG |
|  | Linc01399 | F | CCGAAGCGAATTTGCACCAA |
|  |  | R | ATCCAGGTGGCTACTCGTCT |
|  | Linc00702 | F | AGACGAAGTGCTCCTGATGG |
|  |  | R | TCCATGTGAACACACGCTGA |
|  | FGF7P5 | F | ATGGACACACAATGGAGGGG |
|  |  | R | GCCATAGGAAGAAAGTGGGCT |
|  | HSPA7 | F | AGGATGAAAAGCCCGTGGAA |
|  |  | R | GGCTGAAGCTTCTTGTCGGA |
| ChIP (LINC01189 promoter) | Site 1 | F: -1133 ~ -1153 | TCTTTCTCCCAATGGCCACC |
|  |  | R: -934 ~ -954 | GGTGGGGAGAAAAGAAAGGGA |

**Supplementary Table 6. List of siRNAs for transient transfection**

| **Name** | **siRNA** | **Sense** |
| --- | --- | --- |
| VAX2 | siRNA1 | 5'- CCCAAGCGGACACGUACAUTT -3' |
|  | siRNA2 | 5'- CCAACAUUCUGCGGCUGCUTT -3' |
|  | siRNA3 | 5'- GCAGCUGCAAGAAAGCUAATT -3' |
| LINC01189 | siRNA1 | 5'- AGCUAGAGGUCUAGGAGAAGG -3' |
|  | siRNA2 | 5'- GCAUCUCUGUCAAGUAGUAGA -3' |
|  | siRNA3 | 5'- GAUUCAAGAAGAUGGAUUAAU -3 |
| hnRNPF | siRNA1 | 5'- GGAUGCACAAAGGAAGAAATT -3' |
|  | siRNA2 | 5'- CCCUGUGAGAGUCCAUAUUTT -3' |
|  | siRNA3 | 5'- GCAGCACAGAUAUAUAGAATT -3' |
| Scrambles siRNA | | 5'- UUCUCCGAACGUGUCACGUTT -3' |
