# Supplementary figures and images for: The VAX2-LINC01189-hnRNPF signaling axis regulates cell invasion and migration in gastric cancer
Source: Cell Death Discov. 2023 Oct 21;9:387. doi: 10.1038/s41420-023-01688-4 (PMC10590441; doi:10.1038/s41420-023-01688-4)

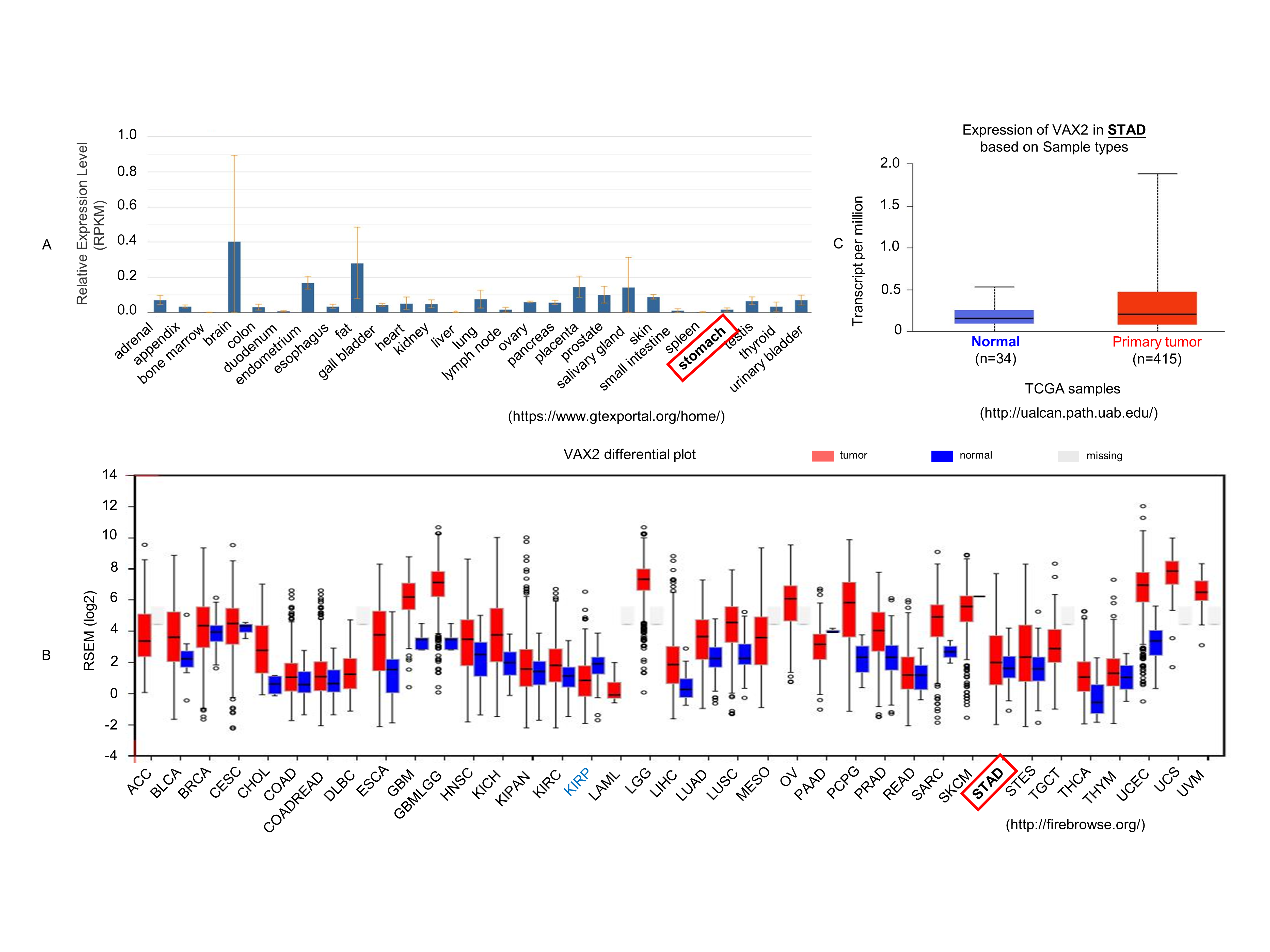

Supplement: Supplementary file 2 — Supplementary Fig. 1 [file 41420_2023_1688_MOESM2_ESM.tif]

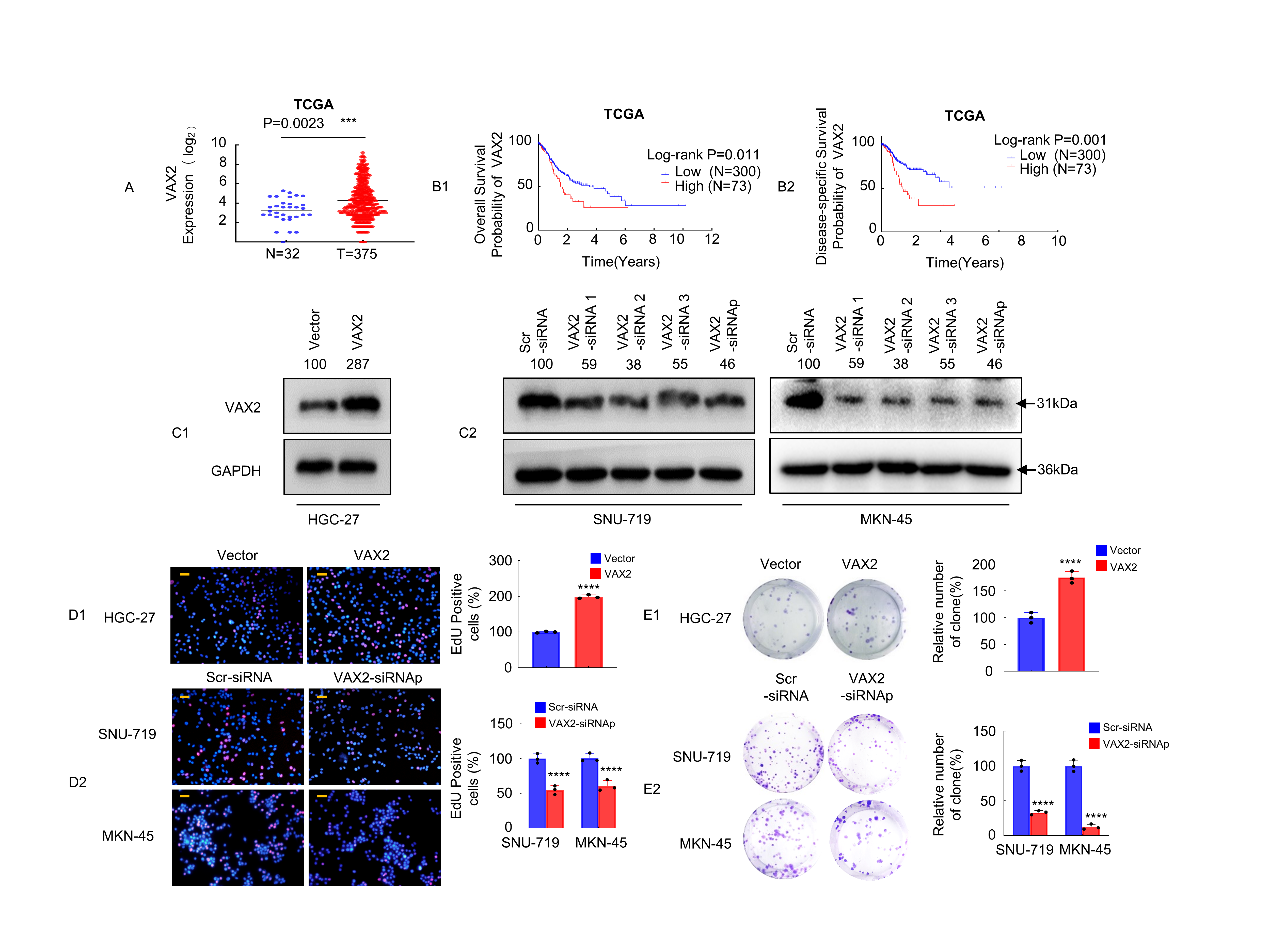

Supplement: Supplementary file 3 — Supplementary Fig. 2 [file 41420_2023_1688_MOESM3_ESM.tif]

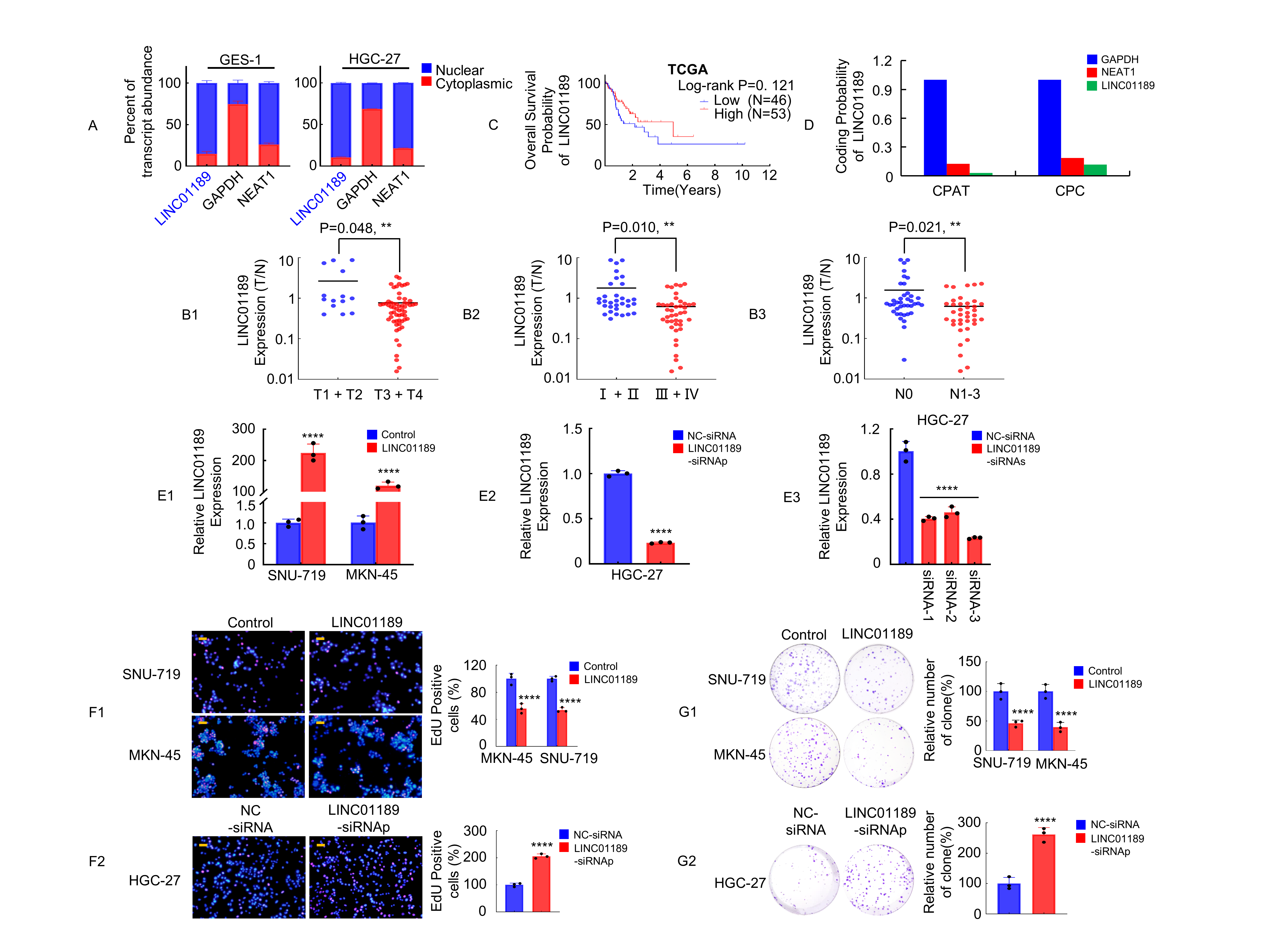

Supplement: Supplementary file 4 — Supplementary Fig. 3 [file 41420_2023_1688_MOESM4_ESM.tif]
