# Supplementary material for: The VAX2-LINC01189-hnRNPF signaling axis regulates cell invasion and migration in gastric cancer
Source: Cell Death Discov. 2023 Oct 21;9:387. doi: 10.1038/s41420-023-01688-4 (PMC10590441; doi:10.1038/s41420-023-01688-4)
Supplement: Supplementary file 5 — Supplementary Figure legends [file 41420_2023_1688_MOESM5_ESM.docx]

**Supplementary Figure legends**

**Supplementary Fig. 1. VAX2 expression in GC tissues is higher than that in normal tissues**. **(A) – (C)** VAX2 expression levels in normal tissues and tumors according to GTEx (A), Firebrowse (B), and UALCAN (C) databases.

**Supplementary Fig. 2. VAX2 is overexpressed in GC and is correlated with malignant biological behavior.** **(A)** Analysis of VAX2 expression in GC patients of TCGA cohort. N, normal individuals; T, patients with tumor. ***, P < 0.01. **(B1/2)** Kaplan–Meier curves for overall survival (OS) (B1) and disease-specific survival (DSS) (B2) by VAX2 expression from TCGA database. Log-rank test. **(C1/2)** Western blotting confirms that overexpression (C1) or knock-down (C2) of VAX2, compared to a negative control (Vector or Scr-siRNA), effects GC cells. siRNAp, siRNA pool. **(D1/2)** The rate of DNA synthesis in GC cells was quantified through EdU incorporation assay. Red fluorescence: EdU-positive cells; Blue fluorescence: total cells. ****, P < 0.001, Vector vs. VAX2; ****, P < 0.001, Scr-siRNA vs. VAX2-siRNAp. siRNAp, siRNA pool. Scale bar: 50 µm. **(E1/2)** The effects of VAX2 overexpression (E1) or VAX2 knock-down (E2) on the proliferation of GC cells was assessed through a colony formation assay. Representative results (left) and quantification (right) are shown. ****, P < 0.001, Vector vs. VAX2; ****, P < 0.001, Scr-siRNA vs. VAX2-siRNAp.

**Supplementary Fig. 3. The subcellular localization, coding ability prediction, expression and functions of LINC01189 in GC. (A)** The subcellular fractionation experiments demonstrated the localization of LINC01189 in GES-1 and HGC-27 cells. Quality control of cytoplasmic RNA or nuclear RNA was conducted using GAPDH or NEAT1. **(B1-3)** LINC01189 expression in the different subgroups stratified by T stage (B1), TNM stage (B2), and N stage (B3). Mann-Whitney U test, **, P < 0.05. **(C)** Correlation between LINC01189 expression and overall survival (OS) in patients with GC using the Kaplan-Meier method based on TCGA database. **(D)** RNA sequences of LINC01189, NEAT1, and GAPDH were evaluated using the Coding Potential Assessment Tool (CPAT) and Coding Potential Calculator 2 (CPC2). **(E1–3)** Expression of LINC01189 as confirmed by qPCR after overexpression (E1) or siRNA/siRNAp knock-down (E2/3) in GC cells. siRNAp, siRNA pool. ****, P < 0.001. Control vs. LINC01189; ****, P < 0.001, NC-siRNA vs. LINC01189-siRNAp; ****, P < 0.001, NC-siRNA vs. LINC01189-siRNAs. **(F1/2)** The rate of DNA synthesis in GC cells was quantified through EdU incorporation assay. ****, P < 0.001, Control vs. LINC01189; ****, P < 0.001, NC-siRNA vs. LINC01189-siRNAp. Scale bar: 50 µm. **(G1/2)** Effects of LINC01189 on the proliferation of GC cell lines as determined by colony formation assay. ****, P < 0.001, Control vs. LINC01189; ****, P < 0.001, NC-siRNA vs. LINC01189-siRNAp.
