# Supplementary material for: The VAX2-LINC01189-hnRNPF signaling axis regulates cell invasion and migration in gastric cancer
Source: Cell Death Discov. 2023 Oct 21;9:387. doi: 10.1038/s41420-023-01688-4 (PMC10590441; doi:10.1038/s41420-023-01688-4)

Fig. 1A
10% GEL

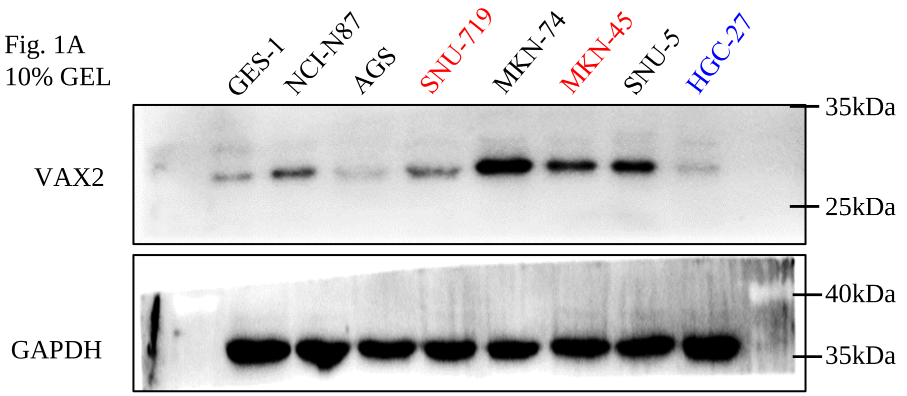

Fig. 1B
10% GEL

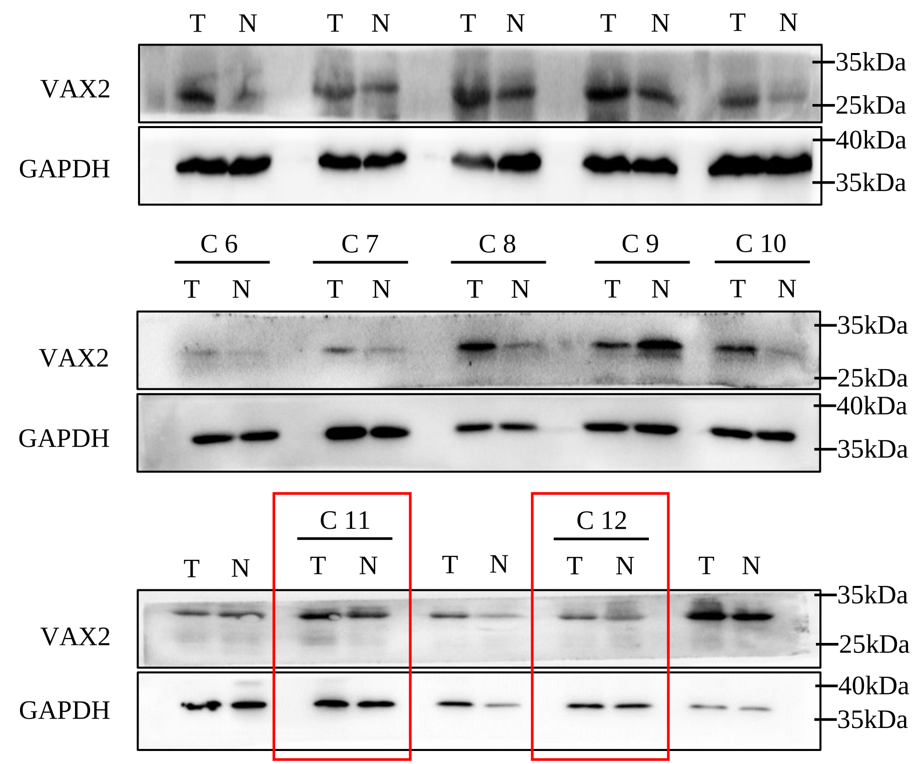

Fig. 5B
10% GEL

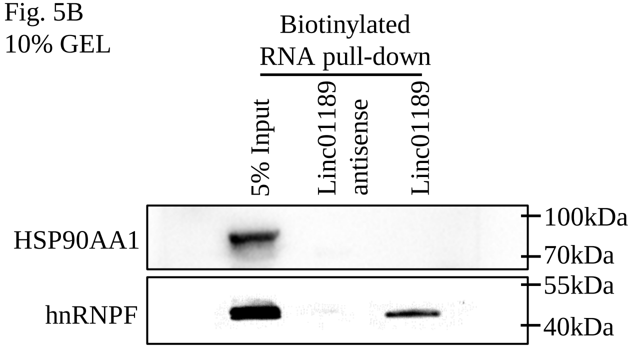

Fig. 5C
10% GEL

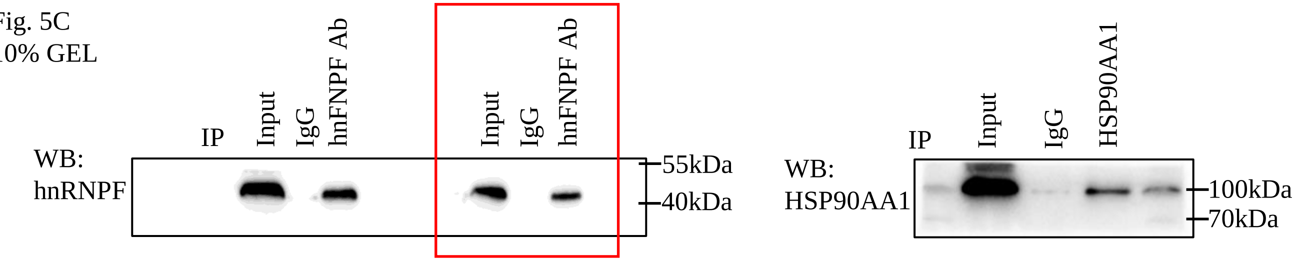

Fig. 5D
10% GEL

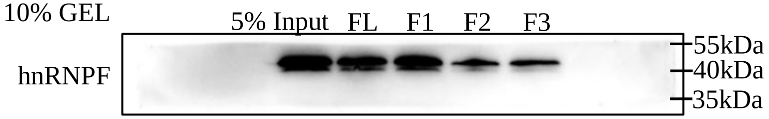

Fig. 5E
8% GEL

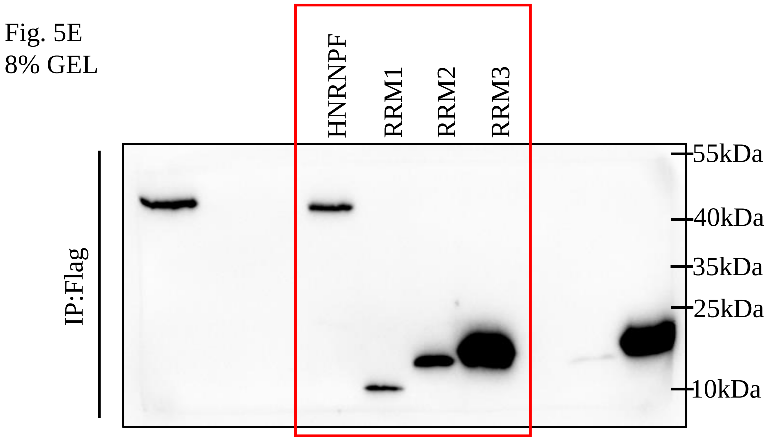

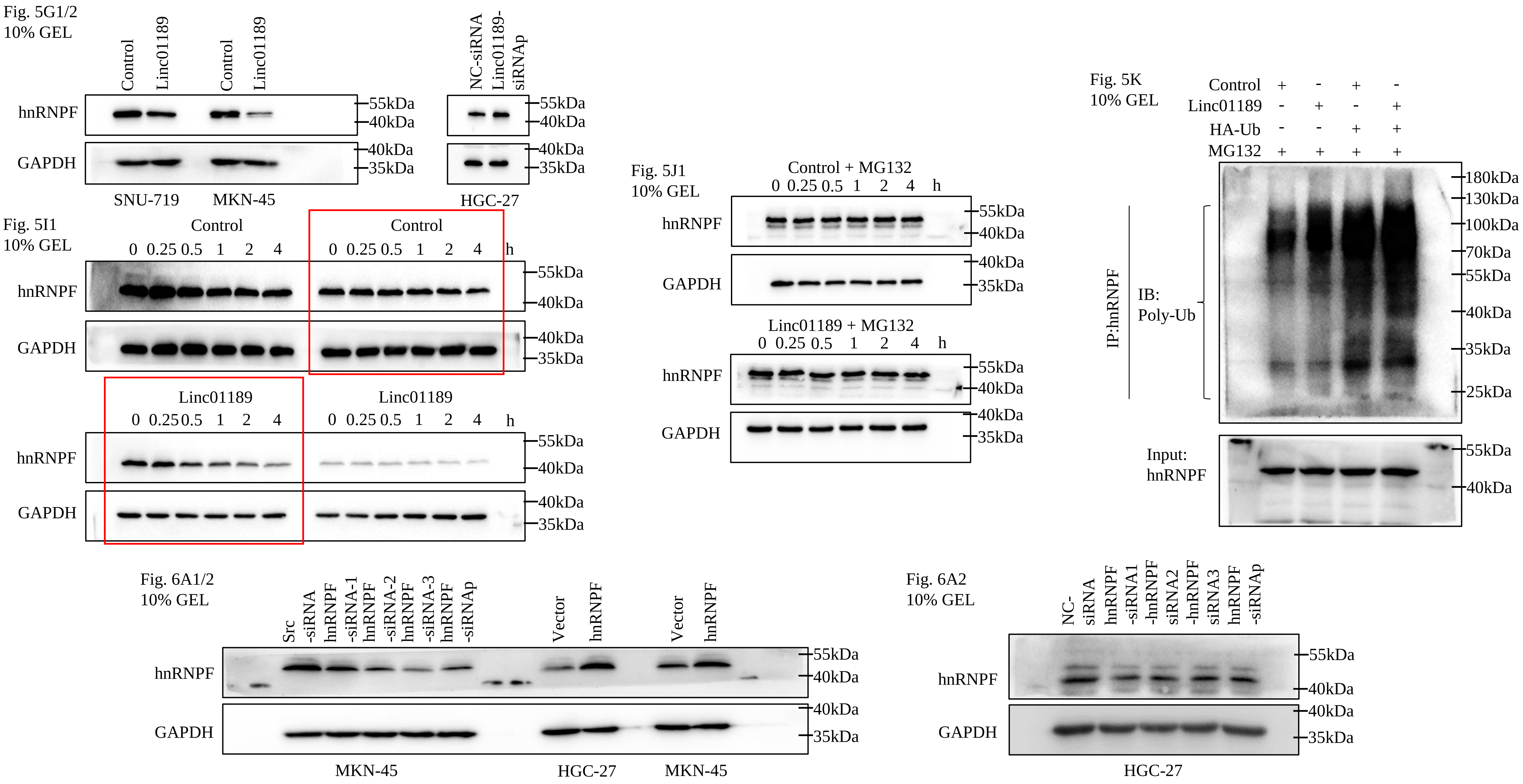

Fig. 5G1/2
10% GEL
Control
Linc01189
Control
Linc01189
NC-siRNA
Linc01189-siRNAp
hnRNPF
GAPDH
55kDa
40kDa
40kDa
35kDa
SNU-719
MKN-45
HGC-27
Fig. 5I1
10% GEL
Control
0 0.25 0.5 1 2 4
Control
0 0.25 0.5 1 2 4 h
hnRNPF
GAPDH
Linc01189
0 0.25 0.5 1 2 4
Linc01189
0 0.25 0.5 1 2 4 h
hnRNPF
GAPDH
Fig. 5J1
10% GEL
Control + MG132
0 0.25 0.5 1 2 4 h
hnRNPF
GAPDH
Linc01189 + MG132
0 0.25 0.5 1 2 4 h
hnRNPF
GAPDH
Fig. 5K
10% GEL
Control + - + -
Linc01189 - + - +
HA-Ub - - + +
MG132 + + + +
IP:hnRNPF
IB: Poly-Ub
180kDa
130kDa
100kDa
70kDa
55kDa
40kDa
35kDa
25kDa
Input: hnRNPF
Fig. 6A1/2
10% GEL
Src
-siRNA
hnRNPF
-siRNA-1
hnRNPF
-siRNA-2
hnRNPF
-siRNA-3
hnRNPF
-siRNAp
Vector
hnRNPF
Vector
hnRNPF
hnRNPF
GAPDH
MKN-45
HGC-27
MKN-45
Fig. 6A2
10% GEL
NC-siRNA
hnRNPF-siRNA1
-hnRNPF siRNA2
-hnRNPF siRNA3
hnRNPF-siRNAp
hnRNPF
GAPDH
HGC-27

Fig. 7A1/2
10% GEL

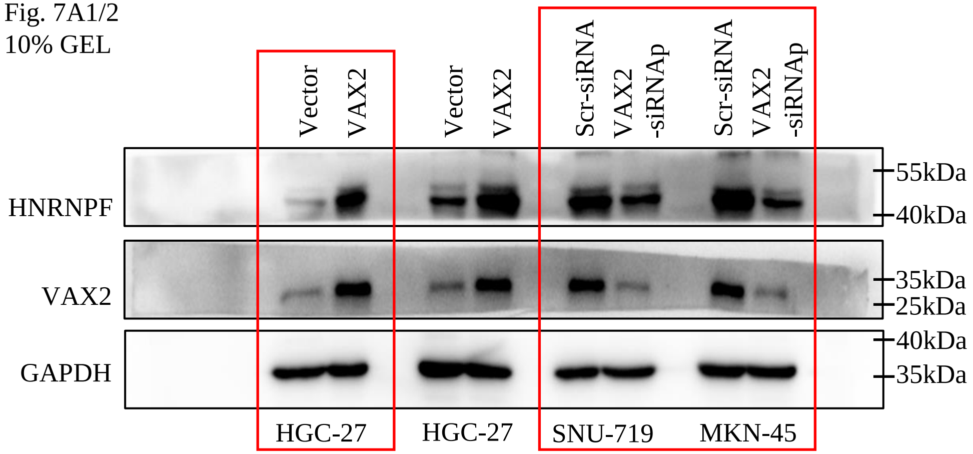

Fig. 7B1/2
10% GEL

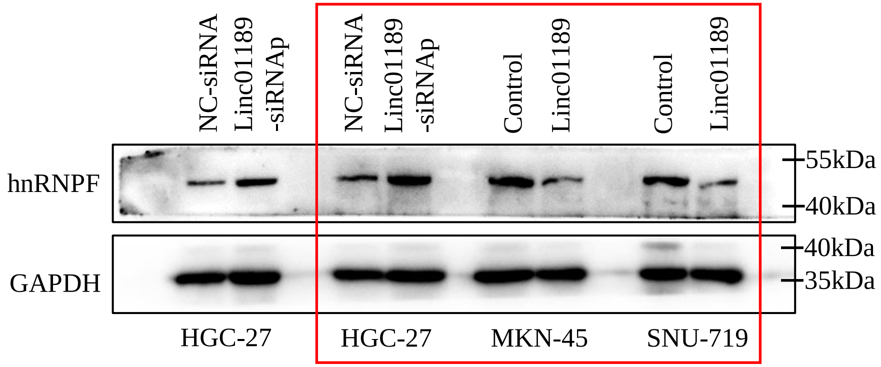

Fig. 7C1
10% GEL

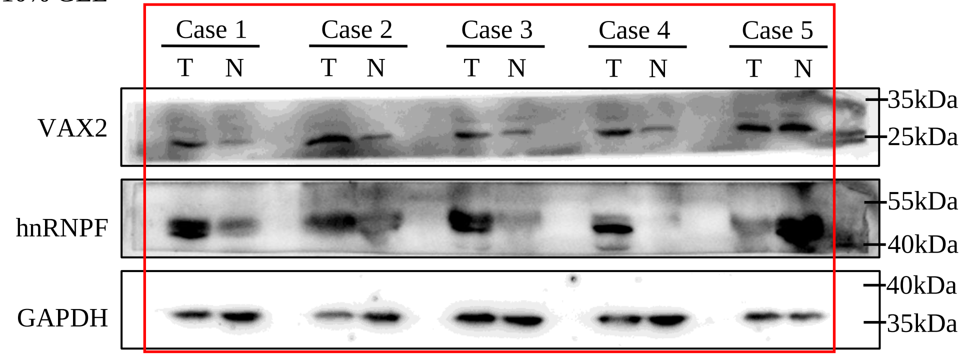

Fig. 7C1
10% GEL

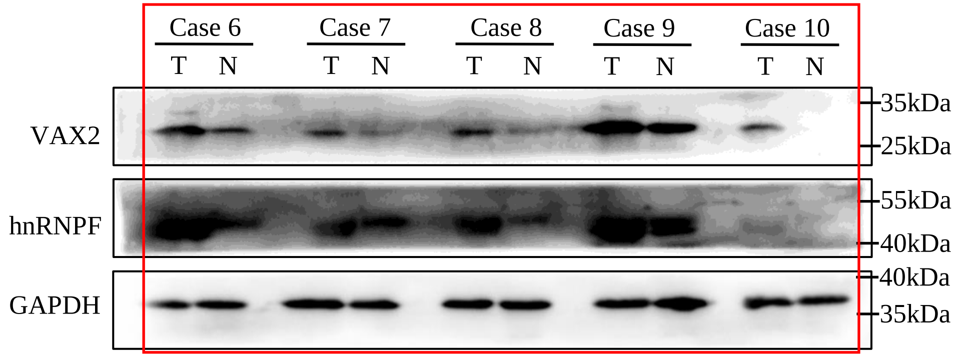

Fig. 7C1
10% GEL

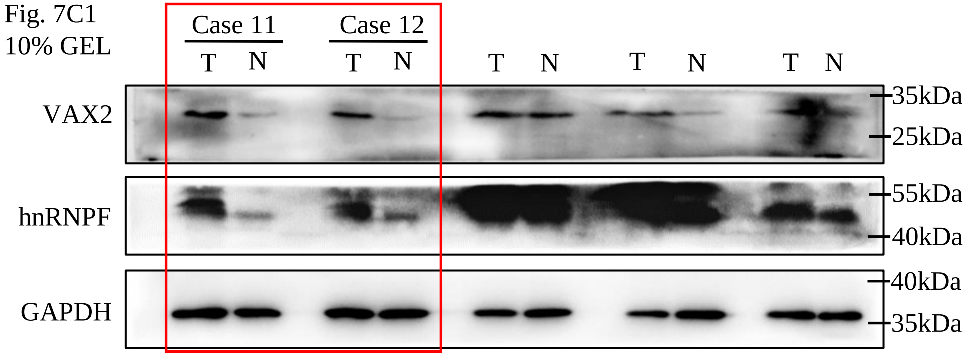

Fig. S2C1/2
10% GEL

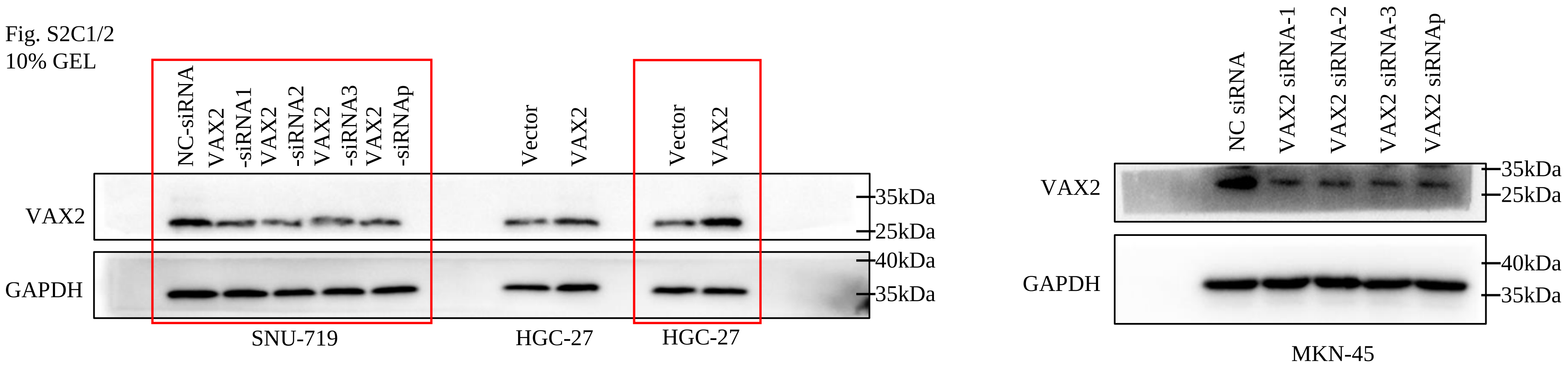

Supplement: Supplementary file 6 — Supplementary File Raw data of western blotting [file 41420_2023_1688_MOESM6_ESM.pdf]
